# CAF Proteins Help SOT1 Regulate the Stability of Chloroplast *ndhA* Transcripts

**DOI:** 10.3390/ijms222312639

**Published:** 2021-11-23

**Authors:** Xiuming Li, Wenzhen Luo, Wen Zhou, Xiaopeng Yin, Xuemei Wang, Xiujin Li, Chenchen Jiang, Qingqing Zhang, Xiaojing Kang, Aihong Zhang, Yi Zhang, Congming Lu

**Affiliations:** 1 State Key Laboratory of Crop Biology, College of Horticulture Science and Engineering, Shandong Agricultural University, Taian 271018, China; lixiuming@sdau.edu.cn; 2 State Key Laboratory of Crop Biology, College of Life Sciences, Shandong Agricultural University, Taian 271018, China; wzluo1998@163.com (W.L.); xiaopengy@126.com (X.Y.); xjli1998@126.com (X.L.); cc_jiang2019@163.com (C.J.); qingqingz2021@163.com (Q.Z.); kxj1201@163.com (X.K.); cmlu@sdau.edu.cn (C.L.); 3 Department of Biology, School of Life Science, Southern University of Science and Technology, Shenzhen 518055, China; zhouw@sustech.edu.cn; 4 Shandong Provincial Key Laboratory of Plant Stress, College of Life Sciences, Shandong Normal University, Jinan 250014, China; wangxuemei2020@sdnu.edu.cn

**Keywords:** chloroplast, *ndhA*, pentatricopeptide repeat protein, RNA stability, RNA splicing

## Abstract

Protein-mediated RNA stabilization plays profound roles in chloroplast gene expression. Genetic studies have indicated that chloroplast *ndhA* transcripts, encoding a key subunit of the NADH dehydrogenase-like complex that mediates photosystem I cyclic electron transport and facilitates chlororespiration, are stabilized by PPR53 and its orthologs, but the underlying mechanisms are unclear. Here, we report that CHLOROPLAST RNA SPLICING 2 (CRS2)-ASSOCIATED FACTOR (CAF) proteins activate SUPPRESSOR OF THYLAKOID FORMATION 1 (SOT1), an ortholog of PPR53 in *Arabidopsis thaliana*, enhancing their affinity for the 5′ ends of *ndhA* transcripts to stabilize these molecules while inhibiting the RNA endonuclease activity of the SOT1 C-terminal SMR domain. In addition, we established that SOT1 improves the splicing efficiency of *ndhA* by facilitating the association of CAF2 with the *ndhA* intron, which may be due to the SOT1-mediated stability of the *ndhA* transcripts. Our findings shed light on the importance of PPR protein interaction partners in moderating RNA metabolism.

## 1. Introduction

Plant cells harbor extranuclear genomes in organelles such as mitochondria and chloroplasts. In land plants, the chloroplast genome carries an average of 120 genes, which encode pivotal components of not only the photosynthetic apparatus but also its transcriptional and translational machineries; therefore, the regulation of gene expression in chloroplasts is particularly important for chloroplast biogenesis [1,2]. Moreover, perturbing the expression of chloroplast genes often severely impairs plant growth and development and can even kill plants [2,3]. 

The proper expression of chloroplast genes requires the import of hundreds of proteins encoded by nuclear genes, thereby creating a coordinated regulation of nuclear and plastid genes [4]. One such example is the nuclear-encoded pentatricopeptide repeat (PPR) family of proteins, characterized by tandem repeats of a degenerate 35–amino acid helical motif [5,6]. Consecutive PPR motifs stack into a right-handed superhelical structure that specifically binds single-stranded RNA in a modular manner [7,8]. PPR proteins function almost exclusively in organellar gene expression, but their number is strikingly expanded in land plants, typically including more than 400 members [5,9,10,11]. The expansion of the PPR family may represent an evolutionary adaption for diverse organellar RNA metabolisms, such as protein-mediated RNA stabilization, RNA cleavage, RNA editing, and RNA splicing [6].

Some PPR proteins harbor a C-terminal small MutS-related (SMR) domain after an array of PPR motifs, and are referred to as PPR-SMR proteins [12,13]. In *Arabidopsis thaliana*, eight PPR-SMR proteins have been identified, with orthologs of all of them present in the major angiosperm clades [12]. Currently, PPR-SMR proteins are attracting extensive attention due to their essential roles in chloroplast retrograde signaling [14], transcription [15,16], and RNA metabolism [17,18,19,20,21]. Recent work from our laboratory demonstrated that the SMR domain of SUPPRESSOR OF THYLAKOID FORMATION 1 (SOT1) has endonuclease activity [22]. The Arabidopsis *sot1* mutant was initially identified as a suppressor of the leaf variegation phenotype of *thf1*, which is not present in *sot1 thf1* double mutants [23]. Both SOT1 and its maize (*Zea mays*) ortholog PPR53 were characterized to promote the maturation of 23S and 4.5S ribosomal RNAs (rRNAs) [23,24]. In addition, our recent findings indicated that the SMR domain of SOT1 cleaves the 23S−4.5S rRNA precursor at the –38 nucleotide upstream of the 5′ end of mature 23S rRNA, and SOT1 facilitates miniribonuclease III–mediated processing during rRNA maturation [22]. The loss of SOT1 or PPR53 results in obvious defects in the expression of the *ndhA* gene, encoding a key subunit of the NADH dehydrogenase-like complex that mediates photosystem I cyclic electron transport and facilitates chlororespiration [23,24,25,26,27]. Based on the classic functions of PPR proteins, Zoschke et al. (2016) suggested that PPR53 regulates the accumulation of *ndhA* transcripts by promoting their stability [24]; however, the binding site of PPR53 is uncertain, and recombinant PPR53 did not bind to *ndhA* transcripts with high affinity, suggesting that the regulatory roles of PPR53 on *ndhA* transcripts requires the participation of other unidentified proteins. The mechanism by which PPR53 or SOT1 participate in stabilizing *ndhA* transcripts thus remains elusive. 

In this study, we explored the biochemical basis of SOT1 in controlling *ndhA* RNA metabolism in chloroplasts. We found that CHLOROPLAST RNA SPLICING 2 (CRS2)-ASSOCIATED FACTOR 1 (CAF1) and CAF2 activate the stabilizing effect of SOT1 on chloroplast *ndhA* transcripts by enhancing the affinity of SOT1 to the 5′ ends of *ndhA* transcripts and inhibiting the RNA endonuclease activity of the C-terminal SMR domain of SOT1. Moreover, we found that SOT1 improves the splicing efficiency of *ndhA* by facilitating the association between CAF2 and its intron, which may be due to the promoted stability of *ndhA* transcripts mediated by the SOT1–CAF interaction complex.

## 2. Results

### 2.1. SOT1 Post-Transcriptionally Regulates ndhA Expression 

**Figure 1:**
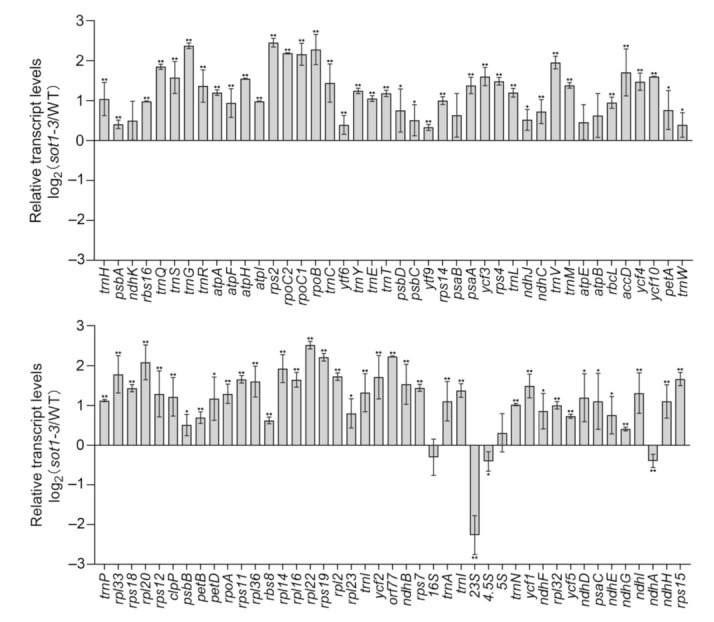
Steady-state levels of chloroplast gene transcripts in the wild type (WT) and *sot1-3*. The transcript levels of the chloroplast genes were determined using quantitative PCR (qPCR) in 12-day-old seedlings. The values are given as log2 fold changes of gene expression in the *sot1-3* mutant relative to that of the WT. Mean values ± SD of three independent experiments are shown. Student’s *t*-test was carried out to determine the significance of the difference of transcript levels between WT and *sot1-3* plants. * indicates a significant difference at *p* < 0.05, whereas ** indicates a significant difference at *p* < 0.01.

To gain a global view of SOT1 in chloroplast RNA metabolism, we performed strand-specific RNA sequencing (RNA-seq) using the total RNA isolated from the 12-day-old wild type (WT) and the *sot1-3* knockout mutant. More than 22 million mappable reads were generated from each sample, of which about 50% aligned to the chloroplast genome (Supplementary Table S1). We calculated the expression levels of each RNA-seq sample as the number of reads per kilobase of transcript per million mapped reads (RPKM) of total mapped reads from the chloroplast, and between replicates, these values were highly reproducible (Supplementary Figure S1). We compared the expression level of the chloroplast genes between the WT and *sot1-3* plants using their RPKM values, revealing that most were increased in the mutant by varying degrees, but the expression levels of *23S rRNA*, *16S rRNA*, *4.5S rRNA*, and *ndhA* were substantially reduced in *sot1-3* (Supplementary Figure S2). This was also confirmed using quantitative PCR (qPCR) to explore the stable transcript levels of 12-day-old WT and *sot1-3* plants, revealing a similar pattern to the RNA-seq findings (Figure 1). These results confirm the previous findings that SOT1 is involved in the regulation of *ndhA* expression and the maturation of 23S and 4.5S rRNA in the chloroplasts [23,24]. Taken together, these results strongly indicate that SOT1 moderates *ndhA* expression at the post-transcriptional level. 

### 2.2. SOT1 Is Required for the Stabilization of the Processed 5′ Ends of ndhA Transcripts

**Figure 2:**
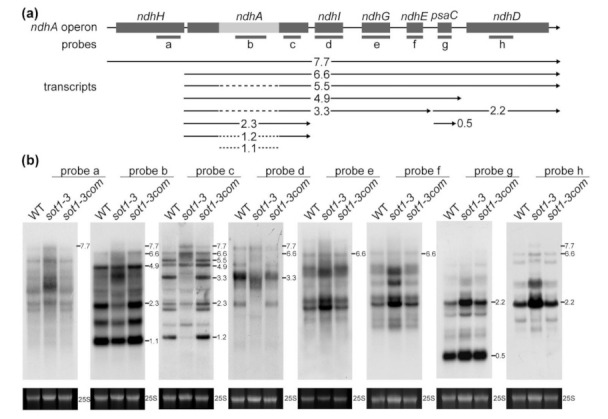
Analysis of transcripts from the *ndhA* operon in the *sot1-3* mutant. (**a**) Schematic illustration of the Arabidopsis *ndhA* operon. *ndhH*, *ndhA*, *ndhI*, *ndhG*, *ndhE*, *psaC*, and *ndhD* are cotranscribed from the *ndhA* operon. The position of each transcript is diagrammed below the operon model, annotated with its length in kilonucleotides. Spliced transcripts are illustrated with a dashed line in the place of the *ndhA* intron. Probes a to h are shown below the operon model. (**b**) RNA gel blot analysis of transcripts from the *ndhA* operon. Ten micrograms of total leaf RNA isolated from 12-day-old wild-type (WT), *sot1-3*, and *sot1-3*com plants was analyzed using an RNA gel blot hybridization. The transcript size is shown in kilonucleotides to the left of each panel. An ethidium bromide staining of the 25S rRNA is shown below each blot as a loading control.

The *ndhA* gene is cotranscribed with *ndhH*, *ndhI*, *ndhG*, *ndhE*, *psaC*, and *ndhD* as a polycistronic operon. This polycistronic operon containing the *ndhA* gene is hereafter referred to as “*ndhA* operon” for convenience. The *ndhA* gene contains a group II intron and is embedded in a polycistronic operon, thereby giving rise to a complex population of processed transcripts (Figure 2a). To date, 10 different transcripts (includes precursors, intermediates, and mature forms) have been identified in maize [24,28], meaning the nascent RNA from the *ndhA* polycistron undergoes complex post-transcriptional processing events to generate mature transcripts.

**Figure 3:**
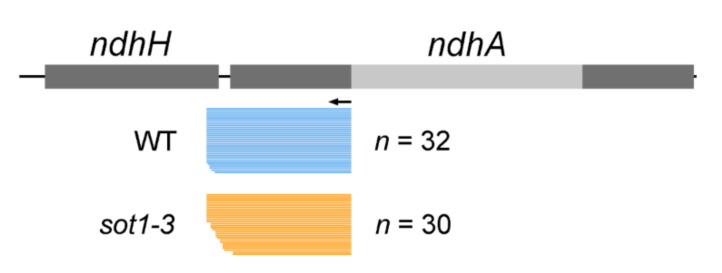
The loss of SOT1 leads to staggered 5′ ends in *ndhA* transcripts. The 5′ ends of *ndhA* transcripts were identified using a 5′-rapid amplification of cDNA ends (RACE). The 5′ ends deduced from the RACE clones in the wild type (WT; blue bars), and *sot1-3* (orange bars) are displayed below the gene model. Each bar represents a single RACE clone.

To characterize the roles of SOT1 in the processing of *ndhA* RNA, we performed an RNA gel blot hybridization to assess the integrity and abundance of transcripts from the *ndhA* operon using different probes positioned in the *ndhA* operon (Figure 2a). Our results showed that several *ndhA* transcript isoforms were missing or reduced in the *sot1-3* mutant but accumulated normally in the WT and complemented *sot1-3* (*sot1-3/35S:SOT1*) plants (Figure 2b). Based on the comparison with the maps of transcripts from the *ndhA* operon, all of the downregulated *ndhA* transcript isoforms had 5′ ends near the *ndhH*–*ndhA* intergenic region (Figure 2a). This result could suggest that SOT1 plays a role in stabilizing the 5′ ends of the *ndhA* transcripts. To test such a possibility, the 5′ ends of the processed *ndhA* transcripts in *sot1-3* were mapped using a 5′-rapid amplification of cDNA ends (5′ RACE) assay with a primer adjacent to the start site of the mature transcripts. The resulting PCR products were in-fusion cloned into the linearized vector then sequenced. The RACE analysis of *sot1-3* showed that the loss of SOT1 resulted in fewer mature *ndhA* transcript isoforms as well as staggered *ndhA* 5′ ends. Among the 30 *ndhA* transcript clones analyzed for the *sot1-3* sample, 16 clones showed shorter 5′ ends than the mature *ndhA* transcripts, whereas 27 of 32 WT clones mapped to the *ndhA* mature ends (Figure 3). Taken together, our results indicate that SOT1 plays an essential stabilizing, processing, or both roles in the *ndhA* transcripts at their 5′ ends.

### 2.3. The SOT1-Mediated Stabilization of ndhA Transcripts Requires the Participation of Other Proteins

**Figure 4:**
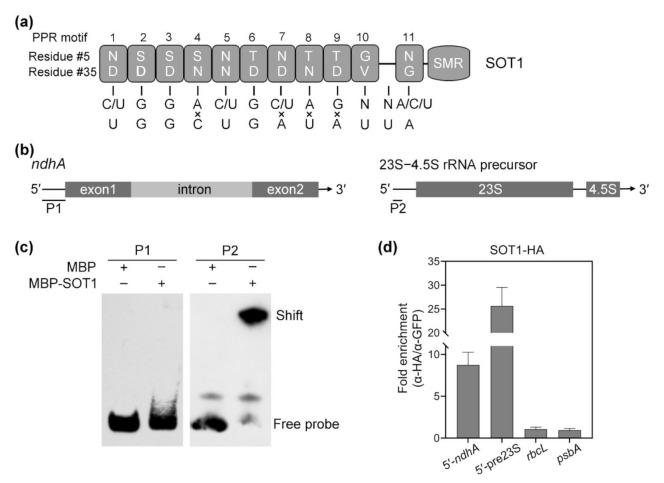
SOT1 binds the 5′ region of the *ndhA* transcripts in vivo. (**a**) Schematic illustration of the PPR motif in SOT1 and its alignment with the predicted binding site in the 5′ region of the *ndhA* transcripts. Each box indicates the amino acid residues at positions 5 and 35 of an individual PPR motif. The potential nucleotide targets of PPR motifs are shown below. (**b**) Schematic representation of the domain structures of *ndhA* mRNA and the 23S−4.5S rRNA precursor. The positions of probes P1 and P2, which were subjected to electrophoretic mobility shift assays (EMSAs), are shown below the models. (**c**) EMSA showing that SOT1 alone exhibits little binding activity to the 5′ ends of *ndhA* transcripts in vitro. A total of 150 nM recombinant MBP and MBP-SOT1 proteins were incubated with 10 nM biotin-labeled probes (P1 and P2). Three independent experiments were performed, and one representative experiment is shown. (**d**) RNA coimmunoprecipitation assays identify that SOT1 binds the 5′ region of *ndhA* transcripts in vivo. The intact chloroplasts were isolated from 12-day-old *sot1-3* complemented plants (*sot1-3/35S:SOT1-HA*). The chloroplast extracts were subjected to immunoprecipitation against HA and GFP antibodies. SOT1 was reported to bind the 5′ ends of the 23S−4.5S rRNA precursor [22,23]; therefore, this interaction served as a positive control. The sample immunoprecipitated with the GFP antibody served as a negative control. The relative RNA-enrichment levels were determined using qPCR. 5′-*ndhA* and 5′-pre23S indicate the 5′ ends of *ndhA* transcripts and 23S−4.5S rRNA precursor, respectively. Mean values ± SD of the triplicate replicates are shown.

Consistent with their function in the regulation of organellar RNA metabolism, a large number of PPR proteins have been shown to bind RNA directly [6,13,29]. Given the results above indicate that SOT1 is involved in the stabilization and splicing of *ndhA* transcripts, the potential targets of SOT1 were examined. Initially, we predicted the binding sequence pattern of SOT1 using the RNA selection “codes” determined by the 5th and 35th residues in each PPR motif, as described previously [7,30,31,32]. SOT1 contains 11 PPR motifs, but the predicted 10th and 11th motifs are separated by a 32 amino acid gap (Figure 4a). This additional ‘motif’ creates a gap that may occupy one base length, and should be taken into account when searching the RNA target for SOT1. Thus, we used the (C/U)GGA(C/U)G(C/U)AGNN(A/C/U) sequence pattern to predict the binding site of SOT1 across the *ndhA* genome sequence, especially the regions close to the transcripts’ termini. We obtained a hit (UGGCUGAUAUUA) containing four mismatches compared to the inquiry sequence adjacent to the 5′ end of the *ndhA* transcripts (Figure 4a). These results suggest that UGGCUGAUAUUA at the 5′ end of *ndhA* transcripts is a potential binding sequence of SOT1.

PPR footprints are the small RNA fragments protected by PPR proteins that stably bind to their target RNAs and can be revealed by deep sequencing [33]. The predicted binding site of SOT1 overlaps with a ‘footprint’ that matches the 5′ end of the *ndhA* transcripts (Figure 4a and Supplementary Figure S3), the abundance of which was significantly reduced in the *sot1-3* mutant compared with the WT [23]. These results show that SOT1 is linked to *ndhA* 5′-localized ‘footprint’ accumulation, further suggesting that SOT1 may bind directly to UGGCUGAUAUUA at the 5′ end of *ndhA* transcripts.

As described above, SOT1 is required for the stabilization of *ndhA* transcripts, so may bind to their 5′ end. A recombinant SOT1 protein was used for electrophoretic mobility shift assays (EMSAs) to further examine the binding of SOT1 to the *ndhA* transcripts. The recombinant SOT1 protein was incubated with a biotin-labeled RNA probe corresponding to the *ndhA* 5′ ends, as well as a probe from the 5′ end of the 23S−4.5S rRNA precursor serving as a positive control (Figure 4b). To our surprise, the slower-migrating band that indicates the formation of the SOT1–RNA complex was only detected using a probe from the 5′ end of 23S−4.5S rRNA precursor but not probes from *ndhA*, including the 5′ end and intron regions (Figure 4c). These results suggest that SOT1 alone exhibits very low binding activity to *ndhA* mRNA.

We next performed an RNA coimmunoprecipitation assay to test whether SOT1 binds such sequences from the *ndhA* transcripts in vivo. We observed an obvious association between SOT1 and the 5′ ends of the *ndhA* transcripts in vivo, but only trace associations with the *rbcL* and *psbA* transcripts served as negative controls (Figure 4b,d). In combination with the above finding that SOT1 alone exhibits no obvious binding activity to *ndhA* transcripts, these results suggest that the binding of SOT1 to the 5′ end of *ndhA* transcripts requires the participation of other proteins.

### 2.4. The CAF Proteins Activate SOT1 to Stabilize ndhA Transcripts by Promoting SOT1 Binding and Inhibiting its RNA Endonuclease Activity

To identify proteins moderating the SOT1-mediated stability of *ndhA* transcripts, we used SOT1 as a bait in a yeast two-hybrid screen and identified two putative interaction partners, CAF1 and CAF2. These proteins, each with two CRM domains, were reported to play essential roles in splicing group II introns in chloroplasts [34,35]. CAF1 and CAF2 bind to CRS2 to form CRS2–CAF1 and CRS2–CAF2 complexes, which regulate the splicing of a set of group II introns in plastid RNAs [36]. Moreover, CRM FAMILY MEMBER 2 (CFM2) resides in large ribonucleoprotein complexes that include CAF1 and/or CAF2 and was shown to promote the splicing of the *ndhA* intron [34]. Thus, we further examined whether SOT1 interacts with these four proteins. 

**Figure 5:**
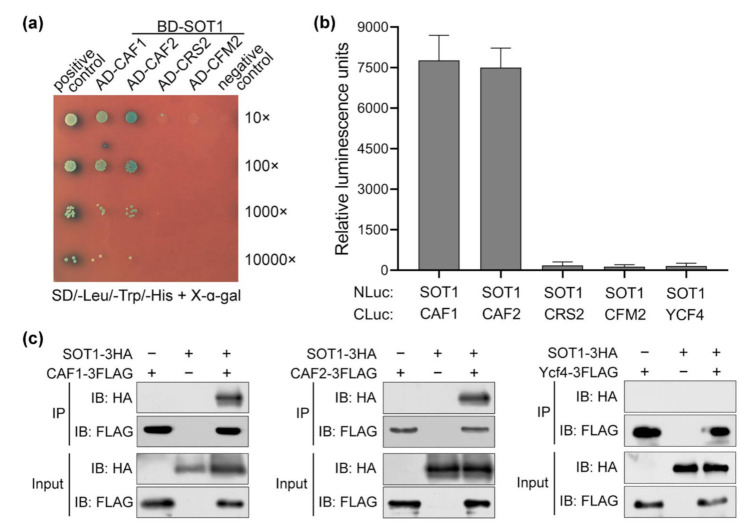
SOT1 directly interacts with CAF1 and CAF2. (**a**) Yeast two-hybrid assays indicate that SOT1 interacts with CAF1 and CAF2. SOT1 was fused to the BD vector; CAF1 (or CAF2, CRS2, and CFM2) was fused to the AD vector. The ability to grow on SD/-Trp-Leu-His-Ade dropout plates indicates an interaction between the two proteins. (**b**) Luciferase complementation assay for interactions between SOT1 and CAF1 versus CAF2. The indicated NLuc and CLuc constructs were transiently coexpressed in *Nicotiana benthamiana* plants through Agrobacterium-mediated transformation, and the luciferase (Luc) activity was measured after 48 h. Error bars indicate SD (*n* = 4). (**c**) Coimmunoprecipitation assays showing the interaction of SOT1 with CAF1 or CAF2. 3 × HA-tagged SOT1 and 3 × FLAG-tagged CAF1 (or CAF2) were coexpressed in Arabidopsis protoplasts. Protein complexes were immunoprecipitated (IP) using an α-FLAG antibody. Immunoblot (IB) analysis of the protein presence in the immunoprecipitates using the α-FLAG and α-HA antibodies, respectively. The assay of interaction between SOT1 and Ycf4 was used as a negative control.

Notably, the yeast two-hybrid assays showed that SOT1 interacted with CAF1 and CAF2, but not with CRS2 or CFM2 (Figure 5a). Next, the interaction of SOT1 with CAF1 and CAF2 was further confirmed using luciferase (Luc) complementation assays. The N-terminal Luc fusion of SOT1 (SOT1-NLuc) and the C-terminal Luc fusion of CAF1, CAF2, CRS2, or CFM2 (CAF1-CLuc, CAF2-CLuc, CRS2-CLuc, or CFM2-CLuc, respectively) were coexpressed in *Nicotiana benthamiana* leaves. Likewise, the coexpression of SOT1-NLuc with CAF1-CLuc or CAF2-CLuc resulted in high levels of Luc activity, but the coexpression of SOT1-NLuc with CRS2-CLuc or CFM2-CLuc resulted in only trace levels of Luc activity similar to the negative control (Figure 5b), confirming the interaction of SOT1 with CAF1 or CAF2. In addition, we coexpressed 3 × HA-tagged SOT1 (SOT1-3HA) and 3 × FLAG-tagged CAF1 or CAF2 (CAF1-3FLAG or CAF2-3FLAG) in Arabidopsis protoplasts and performed coimmunoprecipitation assays. SOT1-3HA was able to precipitate the CAF1-3FLAG and CAF2-3FLAG fusion proteins in vivo (Figure 5c). Our results further demonstrated that SOT1 directly interacts with CAF1 and CAF2 via its 8th and 9th PPR motifs (Supplementary Figure S4). 

**Figure 6:**
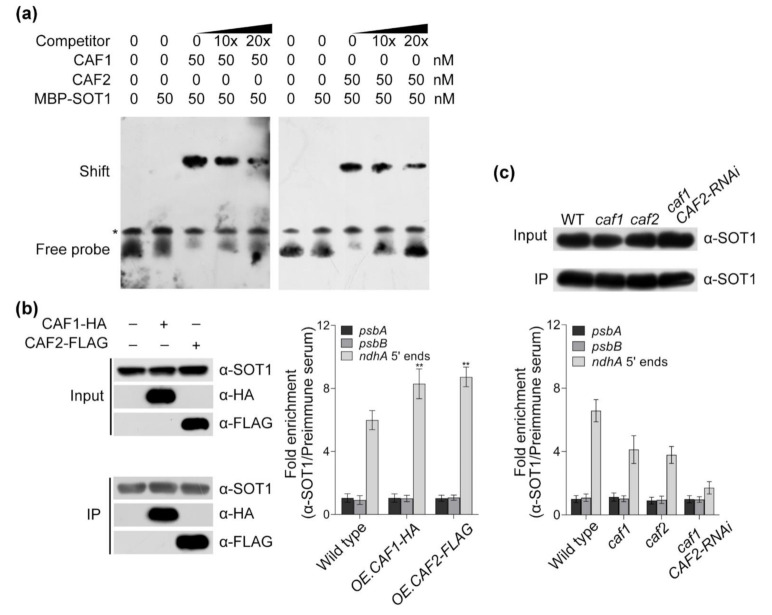
CAF1 and CAF2 promote the binding of SOT1 to the 5′ end of *ndhA* transcripts. (**a**) Electrophoretic mobility shift assay (EMSA) showing the positive effect of CAF1 and CAF2 on the binding of SOT1 to the 5′ end of *ndhA* transcripts. The predicted binding sequence (UGGCUGAUAUUA) of SOT1 on *ndhA* transcripts was synthesized and labeled with biotin. Increasing concentrations of recombinant CAF1 or CAF2 proteins were incubated with 10 nM biotin-labeled probe and the MBP-SOT1 protein. The competitor used in the competition experiments was an unlabeled probe corresponding to the predicted binding sequence of SOT1 on the *ndhA* transcripts. The asterisk indicates an unspecific band. Three independent experiments were performed, and one representative experiment is shown. (**b**) Overexpressing CAF proteins promoting the binding of SOT1 to the 5′ end of *ndhA* transcripts in vivo. The HA-tagged CAF1 and FLAG-tagged CAF2 were overexpressed (OE) in protoplasts isolated from 12-day-old seedlings. Chloroplasts were harvested from the transfected protoplasts. Protein complexes were immunoprecipitated (IP) using an α-SOT1 antibody. (Left) Immunoblot analysis of the protein presence in chloroplast extract as immunoprecipitated using an α-SOT1 antibody. The immunoblot results showed a comparable enrichment of SOT1 protein in samples. (Right) The relative levels of *psbA*, *psbB*, and *ndhA* 5′-end mRNAs in the α-SOT1 immunoprecipitated complexes were determined using a qPCR. Student’s *t*-test was carried out to determine the significance of the fold enrichments of SOT1 on *ndhA* 5’-end RNAs between WT and OE.CAF protoplast. ** indicates a significant difference at *p* < 0.01. Mean values ± SD of the triplicate replicates are shown. (**c**) The loss of CAF proteins leads to the decreased association of SOT1 with the 5′ end of *ndhA* transcripts. The stromal extracts from wild-type, *caf1*, *caf2*, and *caf1 CAF2*-interference (RNAi) plants were immunoprecipitated with an α-SOT1 antibody. (Upper) Immunoblot analysis of the protein presence in the chloroplast extract, as immunoprecipitated using an α-SOT1 antibody. (Lower) The relative levels of *psbA*, *psbB*, and *ndhA* 5′-end mRNAs in the α-SOT1 immunoprecipitated complexes were determined using qPCR. Mean values ± SD of the triplicate replicates are shown.

The above results suggest that the association of SOT1 with *ndhA* transcripts is facilitated by other proteins; therefore, we next performed EMSAs to investigate whether CAF1 and/or CAF2 promoted SOT1 binding to the 5′ end of *ndhA* transcripts. Our results showed that the affinity of SOT1 to the potential binding sequence, revealed by the “PPR code ” prediction, was considerably enhanced after the addition of recombinant CAF1 or CAF2 proteins (Figure 6a). Considering SOT1 directly interacts with CAF1 or CAF2, they may form an interaction complex showing a high affinity to the 5′ end of *ndhA* transcripts. Next, we overexpressed the CAF1 and CAF2 proteins in 12-day-old WT protoplasts and performed an RNA coimmunoprecipitation assay to determine the association of SOT1 with the 5′ end of the *ndhA* transcripts. When CAF1 or CAF2 protein was overexpressed in the Arabidopsis protoplast system, the association levels of SOT1 with the *ndhA* 5′ ends were significantly increased by about 40% compared with those in the WT (Figure 6b). In addition, the loss of CAF1 and/or CAF2 resulted in a considerable decrease in the association of SOT1 with the *ndhA* 5′-end transcripts (Figure 6c). Taken together, these results showed that both CAF1 and CAF2 are capable of promoting SOT1 binding to the 5′ end of *ndhA* transcripts to enhance the stabilizing role of SOT1 in *ndhA* transcripts.

**Figure 7:**
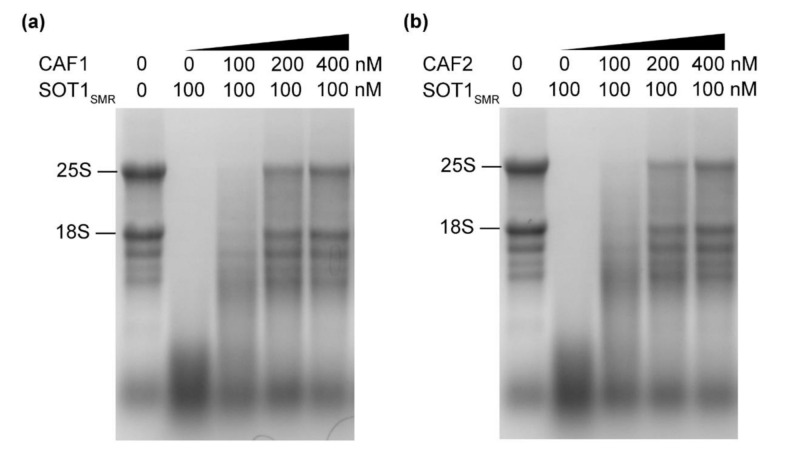
The RNA nuclease activity of the SMR domain of SOT1 can be inhibited by CAF proteins. The recombinant SOT1_SMR_ protein, together with increasing concentrations of CAF1 (**a**) or CAF2 (**b**) proteins, was incubated with total wild-type Arabidopsis RNA at 25 °C for 30 min. The reaction products were separated on agarose/formaldehyde gels and observed using ethidium bromide staining. Three independent experiments were performed, and one representative experiment is shown.

SOT1 contains a C-terminal SMR domain after its P-class PPR motifs [13]. Previous research from our laboratory revealed that the SMR domain of SOT1 has RNA endonuclease activity and cleaves the 23S−4.5S rRNA precursor to promote the maturation of 23S and 4.5S rRNA [22]. In general, the RNA endonuclease activity of the SMR domain of SOT1 (SOT1_SMR_) is unfavorable for stabilizing *ndhA* transcripts. Given that SOT1_SMR_ interacted with CAF1 and CAF2 (Figure 5 and Supplementary Figure S4), we deduce that CAF1 and/or CAF2 plays a role in regulating the RNA nuclease activity of SOT1_SMR_. To investigate the potential roles of the CAF proteins, we set out to examine the RNA nuclease activity of SOT1_SMR_ in the presence and absence of recombinant CAF proteins using Arabidopsis total RNA. We observed a reduced and smeared rRNA band on the RNA gel, indicating that the total RNA was degraded by SOT1_SMR_. This shows that SOT1_SMR_ has RNA nuclease activity that can efficiently cleave Arabidopsis total RNA. In contrast, the rRNA integrity was considerably improved after the addition of recombinant CAF1 or CAF2, demonstrating that the RNA degradation by SOT1_SMR_ was reduced (Figure 7). These results indicate that both CAF1 and CAF2 have the ability to restrain the SMR domain of SOT1 at the RNA endonuclease activity level, and therefore enhance the role of SOT1 in stabilizing *ndhA* transcripts.

### 2.5. SOT1 Promotes the Splicing of ndhA Transcripts

**Figure 8:**
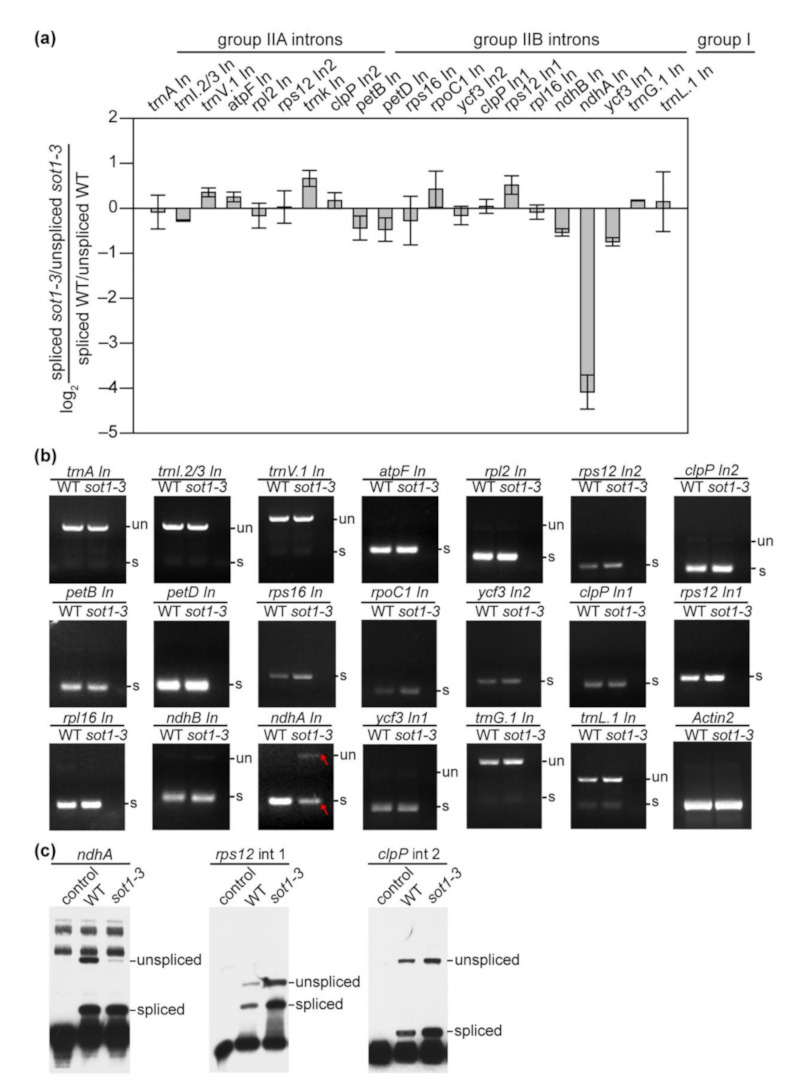
SOT1 is required for the splicing of the *ndhA* intron. (**a**) qPCR analysis of the splicing efficiency of chloroplast introns. The values represent the difference in the ratios of spliced to unspliced transcripts in 12-day-old *sot1-3* seedlings compared with the wild type. Four biological replicates were analyzed. Error bars indicate SD. (**b**) Reverse transcription (RT)-PCR analysis of all intron-containing plastid genes in total leaf RNA from the WT and *sot1-3*. Primers flanking each plastid intron were used, as shown in Supplementary Table S2. The bands of *ndhA* intron in *sot1-3* are highlighted in red. un, unspliced transcripts; s, spliced transcripts. (**c**) Poisoned primer extension analysis of the splicing efficiency of *ndhA*. Biotin-labeled DNA primers complementary to the exon sequences near the splice junction were used to initiate reverse transcription in the presence of a dideoxynucleotide that terminates the extension after different distances on spliced and unspliced transcripts. The poisoned primer extension assays for *rps12* intron 1 and *clpP* intron 2 were used as controls.

Considering the group II intron within *ndhA* transcripts locates about 600 nucleotides downstream of their 5′ ends, which are stabilized by SOT1–CAF interaction complexes, we next determined whether SOT1 moderates the splicing efficiency of *ndhA*. Indeed, we found that the amount of *ndhA* intron excised was reduced in the *sot1-3* mutant compared with the WT, with the spliced transcript isoforms showing a larger decrease than the unspliced isoforms (Figure 8a). These results suggest that SOT1 is involved in *ndhA* intron splicing. To verify this possibility, we conducted qPCR and RNA-seq to calculate the ratio of spliced to unspliced transcripts, which is an indicator of splicing efficiency, in the WT and *sot1-3* plants. Indeed, there was a considerable decrease in the splicing efficiency of the *ndhA* intron in the mutant, but no obvious changes in that of the other intron-containing chloroplast genes (Figure 8a and Supplementary Figure S5). Similar results were also observed using a reverse transcription (RT)-PCR assay, followed by resolving the spliced and unspliced products on agarose gels (Figure 8b). Moreover, a poison primer extension analysis was conducted to detect the splicing efficiency of *ndhA*, *rps12* intron 1, and *clpP* intron 2, with the result consistent with those of the PCR and RNA-seq analyses (Figure 8 and Supplementary Figure S5). These results show that the efficiency of intron splicing in *ndhA* is reduced in *sot1-3* compared with the WT.

### 2.6. The Association of CAF2 with the ndhA Intron Is Decreased in sot1-3

As described above, SOT1, a PPR protein, is required for the splicing of the *ndhA* intron. Since PPR proteins normally bind to their target RNAs, we tried to determine whether SOT1 could also bind to the *ndhA* intron. We first conducted an EMSA to examine the binding of a recombinant SOT1 protein to various biotin-labeled RNA probes corresponding to the *ndhA* intron. This revealed that SOT1 does not bind directly to the *ndhA* intron (Supplementary Figure S6a,b). Next, we performed an RNA coimmunoprecipitation assay to test whether SOT1 binds the *ndhA* intron in vivo, but no association was observed between them (Supplementary Figure S6a,c). Since SOT1 has been demonstrated to bind the 5′ end of *ndhA* transcripts, these results suggest SOT1 promotes *ndhA* splicing through an indirect role.

**Figure 9:**
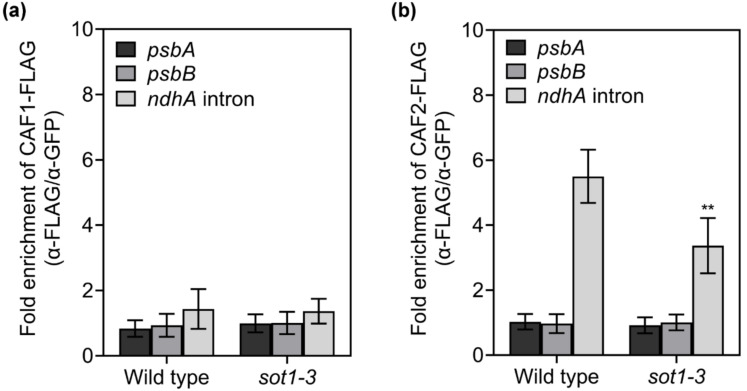
The loss of SOT1 results in the decreased association of CAF2 with the *ndhA* intron. The C-terminal FLAG fusion of CAF1 or CAF2 was expressed in protoplasts isolated from 12-day-old WT and *sot1-3* seedlings. Chloroplasts were harvested from the transfected protoplasts. The relative transcript levels of the *psbA*, *psbB*, and *ndhA* introns in α-FLAG immunoprecipitated complexes were determined via qPCR. Student’s *t*-test was carried out to determine the significance of the fold enrichments of CAF2 on *ndhA* intron between WT and *sot1-3* plants. ** indicates a significant difference at *p* < 0.01. Mean values ± SD of the triplicate replicates are shown.

The basic function of the CAF proteins is as general splicing factors for chloroplast genes. A mild defect in *ndhA* splicing was previously reported in *caf1* mutants, while *ndhA* splicing was nearly abolished in the *caf2* mutants, suggesting that the splicing of the *ndhA* intron is weakly dependent on CAF1 but strongly dependent on CAF2 [37,38]. The CAF proteins play a pivotal role in the splicing of chloroplast group II introns. They bind CRS2 to form the stable CRS2–CAF1 and CRS2–CAF2 complexes, respectively, via a 22–amino acid motif in the COOH-terminal region of the CAF proteins [36]. These complexes have high affinities to their cognate group II introns in vivo, with the CAF subunit determining the intron specificity of the complex [39]. Since the splicing efficiency of *ndhA* was considerably decreased in *sot1-3*, we examined the association of CAF1 and CAF2 with the *ndhA* intron in WT and *sot1-3* plants via an RNA coimmunoprecipitation assay. We found that trace amounts of the *ndhA* intron coimmunoprecipitated with CAF1 from the chloroplast protein extract (Figure 9), which is consistent with the result reported previously in maize [40]. By contrast, the *ndhA* intron strongly coimmunoprecipitated with CAF2 from the chloroplast protein extract from the WT, but much less *ndhA* intron precipitated with CAF2 in *sot1-3* than in the WT (Figure 9). In addition, we tried to complement *sot1-3* plants with the PPR domain alone (*sot1-3*/*35S:SOT1-PPR*). The defect in *ndhA* splicing was rescued in the *sot1-3*/*35S:SOT1-PPR* plants (Supplementary Figure S7), confirming the regulatory roles of SOT1 in *ndhA* splicing. These results suggest that SOT1 regulates the splicing of the *ndhA* intron by facilitating the association of CAF2 with the *ndhA* intron region.

## 3. Discussion

How does SOT1 regulate the stability of *ndhA* transcripts? SOT1 binds the 5′ ends of *ndhA* transcripts in vivo, but the binding site between SOT1 and the *ndhA* transcripts contains four mismatched nucleotides when compared with the theoretical binding sequence, as revealed using the “PPR codes” prediction. These mismatched nucleotides within the *ndhA*–SOT1 binding site decrease the binding affinity of recombinant SOT1 to a trace level in vitro (Figure 4), indicating that the binding of SOT1 to the 5′ ends of the *ndhA* transcripts requires the participation of additional proteins. Indeed, we found that the CAF proteins (CAF1 and CAF2) interact with SOT1 via its PPR motifs 8 and 9, as well as the SMR domain, which would improve the RNA-binding affinity of SOT1 to 5′ ends of *ndhA* transcripts (Figure 5 and Figure 6, and Supplementary Figure S4). A previous study found that the plastid editing factor MORF9 could interact with an artificial PLS-type PPR protein and increase the RNA-binding activity of this PPR protein to its target RNA. The crystal structures of MORF9, artificial PPR, and MORF9-bound PPR binary complexes reveal the conformational changes that take place in the complex, explaining the molecular mechanisms by which MORF9 induces PLS-type PPR protein binding to its target RNA [41]. On the basis of this finding, we suggest that the interaction between the CAF proteins and SOT1 may induce conformational changes in SOT1 to improve its binding to the mismatched nucleotides within the *ndhA*–SOT1 binding site at the 5′ end of the *ndhA* transcripts. In addition, SOT1 contains a C-terminal SMR domain with RNA endonuclease activity [22]. In general, the RNA endonuclease activity of the SMR domain of SOT1 would have an adverse effect on stabilizing *ndhA* transcripts; however, we found that the CAF protein can also restrict this RNA endonuclease activity in SOT1 (Figure 7). CAF proteins, therefore, likely regulate SOT1 activity similarly to other PPR proteins lacking an SMR domain, stabilizing *ndhA* transcripts by protecting them from 5′ → 3′ exonucleolytic degradation. Since most of *ndhA* precursor and mature isoforms were missing or reduced in the *sot1-3* mutant but accumulated normally in the WT (Figure 2), these findings also highlight the novel functions of CAF proteins in maintaining the stability of *ndhA* transcripts, including both spliced and unspliced transcripts.

RNA splicing represents an essential step for chloroplast gene expression, in which the introns from pre-mRNAs are removed and exons are joined together to enable the production of mature mRNAs containing the correct genetic information. Based on the primary and secondary structures, as well as the splicing mechanisms, the introns are classified into two major families, group I and group II; *ndhA* contains one group II intron [24,39]. The splicing efficiency of *ndhA* was considerably decreased in *sot1-3* compared with that of the WT (Figure 8), suggesting SOT1 harbors a regulatory role in the *ndhA* splicing process. A puzzling question is how SOT1 also regulates the splicing of the *ndhA* intron. Its lack of an evident binding affinity to the *ndhA* intron (Supplementary Figure S6) suggests that SOT1 indirectly enhances *ndhA* splicing. The basic function of CAF proteins is to serve as an essential general splicing factor for group II introns. The CAF proteins bind to CRS2 to form the CRS2–CAF1 and CRS2–CAF2 complexes via a 22–amino acid motif in the COOH-terminal region of the CAF proteins [37,38]. These complexes possess high affinity to their cognate group II introns, with the CAF subunit in the complex determining its specificity to introns [37,38]. Our results demonstrate that the loss of SOT1 results in a considerable decrease in the occupancy of CAF2 on the *ndhA* intron region (Figure 9), suggesting that SOT1 regulates *ndhA* splicing by facilitating the association of CAF2 with the *ndhA* intron. In addition, we attempted to complement *sot1-3* plants with the PPR domain alone. The defect in *ndhA* splicing was rescued in the *sot1-3*/*35S:SOT1-PPR* plants (Supplementary Figure S7), suggesting that the SOT1-mediated *ndhA* transcript stability may play a role in regulating *ndhA* splicing. We, therefore, propose that this SOT1-mediated stability ensures the production of mature *ndhA* 5′ ends rather than the formation of staggered 5′ ends that can be degraded by the 5′ → 3′ exonuclease. Mature *ndhA* 5′ ends would also help the *ndhA* intron fold into a proper RNA structure to be targeted by the general splicing factor CAF2 or other factors. Alternatively, as tertiary interactions of intron-binding sites (IBS1 and IBS2) in exon 1 with exon-binding sites (EBS1 and EBS2) in the intron are necessary for splicing [39,42], the defect of SOT1-mediated stability may interfere with the pairing of IBS-EBS and impair the splicing of the *ndhA* intron.

Based on the phylogenetic analysis, SOT1 and PPR53 are considered orthologous proteins. In maize, the loss of PPR53 leads to defects in the maturation of 23S and 4.5S rRNAs, as well as the reduced stabilization of *ndhA* transcripts [24]. We performed RNA-seq to gain a global view of SOT1 in the chloroplast RNA metabolism in Arabidopsis. In addition to its PPR53-like functions, SOT1 was also found to promote the splicing of the *ndhA* intron (Figure 8 and Supplementary Figure S5); therefore, it appears that the functions of SOT1 and its ortholog PPR53 have not been strictly conserved. SOT1 evolved an additional function in regulating *ndhA* intron splicing in Arabidopsis, reflecting a fast-evolving RNA metabolism in the chloroplasts of land plants. On the other hand, in model species of barley, Arabidopsis, and maize, all the *ndhA* isoforms showed the same 5′-end 65 or 66 nucleotides upstream of the *ndhH* stop codon [23,28,43], suggesting the 5′ ends of *ndhA* transcript are conserved across monocots and dicots, and also indicating a conserved role of SOT1 orthologs in *ndhA* 5′-ends stabilization.

**Figure 10:**
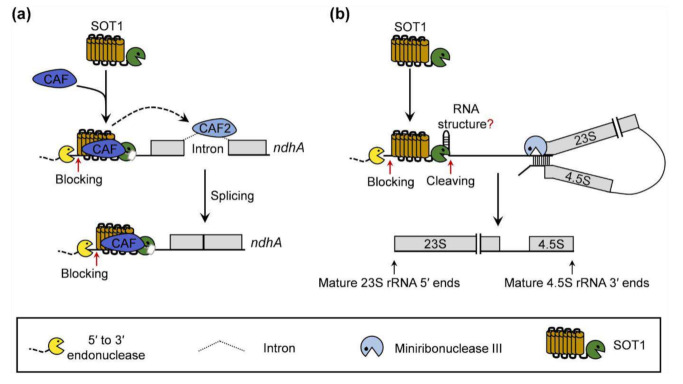
Proposed model for the role of SOT1 in RNA metabolism in the chloroplasts. (**a**) SOT1 functions together with the CAF proteins (CAF1 or CAF2) and forms an interaction complex that synergistically promotes SOT1 binding to the 5′ ends of *ndhA* transcripts, thereby blocking the 5′ to 3′ exonuclease invasion. The CAF proteins are capable of inhibiting the RNA endonuclease activity of the SOT1 SMR domain, which facilitates the stabilizing effect of SOT1 on the *ndhA* transcripts. In addition, SOT1 helps promote the splicing of the *ndhA* precursor by facilitating the association of CAF2 with the *ndhA* intron region. Red arrows indicate the blocking sites for exonucleases. (**b**) Without interacting with the CAF proteins, SOT1 directly binds to the 5′ ends of the chloroplast 23S−4.5S rRNA precursor via its PPR motifs, which block 5′ to 3′ exonuclease invasion. The C-terminal SMR domain of SOT1 cleaves the rRNA precursor at the −38 nt site relative to the 5′ end of mature 23S rRNA. SOT1 also facilitates the processing by miniribonuclease III during the maturation of 23S and 4.5S rRNA [13,22,23]. Red arrows indicate the blocking sites for exonucleases and cleaving site of SOT1, respectively. The question mark indicates that the RNA structure formed around the SOT1 cleaving site is speculated.

In summary, based on the knowledge gained in this study as well as the previous reports on the roles of SOT1 in rRNA maturation [22,23,24], we propose a working model for SOT1 in the chloroplast RNA metabolism. SOT1 interacts with CAF proteins (CAF1 or CAF2) to form an interaction complex that confers high binding affinity to the 5′ ends of *ndhA* transcripts. The CAF proteins also inhibit the RNA endonuclease activity of the SMR domain in SOT1. Thus, the CAF proteins make SOT1 behave similarly to other PPR proteins lacking the SMR domain, stabilizing *ndhA* transcripts to protect them from 5′ → 3′ exonucleolytic degradation. In addition, the SOT1-mediated stability of *ndhA* may help promote the splicing of *ndhA* precursors by facilitating the association of CAF2 with the *ndhA* intron (Figure 10a). Without interacting with CAF proteins, SOT1 also directly binds the 5′ ends of 23S−4.5S rRNA precursor via its PPR motifs and cleaves it at the −38 nt site relative to the 5′ ends of mature 23S rRNA via its C-terminal SMR domain, facilitating the proper 5′ maturation of 23S rRNA and 3′ maturation of 4.5S rRNA by miniribonuclease III (Figure 10b).

## 4. Materials and Methods

### 4.1. Plant Materials and Growth Conditions

The *Arabidopsis thaliana* (Col-0) T-DNA insertion mutants *caf1* (SALK_025042) and *caf2* (SALK_008478) were obtained from the Arabidopsis Biological Resource Center (ABRC, Columbus, OH, USA) (http://abrc.osu.edu/; accessed on 12 December 2013). The mutant *sot1-3* line was isolated from a pSKI15 T-DNA-mutagenized *Arabidopsis thaliana* library (ecotype Col-0) based on its pale-green leaf phenotype [22]. The mutant plants were backcrossed three times to WT plants. The *sot1-3*-complemented lines were generated by introducing the *SOT1* coding sequence, under the control of the 35S promoter, into the *sot1-3* mutant using the vector PBI121-HA.

The seeds of the WT, mutants, and sot1-3 complemented plants were sur-face-sterilized and sown on Murashige and Skoog (MS) medium containing 2% (*w/v*) sucrose and 0.4% (*w/v*) gellan gum (Coolaber, Beijing, China). The seedlings were grown on MS me-dium or soil under long-day conditions (14 h light/10 h dark) with a photon flux den-sity of 80 μmol m^−2^ s^−1^ at 22 °C.

### 4.2. Strand-specific RNA Sequencing

Strand-specific RNA sequencing was performed according to Hotto et al. (2015) [44]. Two independently replicated experiments were performed. Total RNA was isolated from 12-day-old WT and *sot1-3* seedlings. After being treated with DNase (Promega, Madison, WI, USA), a RiboMinus plant kit (ThermoFisher, Waltham, MA, USA) to the RNA to remove rRNA. The RNA-seq library was prepared from the rRNA-depleted DNA-free RNA by OE Biotech. The samples were run on a single lane using an Illumina HiSeq 2500 with 150-bp pair-end reads. After quality control, strand-specific reads with a quality higher than 30 and a length longer than 60 were aligned to the Arabidopsis chloroplast genome (TAIR10) using TopHat2. The relative transcript abundance and splicing efficiency of the chloroplast genes were calculated using the ChloroSeq pipeline [45].

### 4.3. RNA Preparation, qPCR, and Gel Blot Analysis

The total RNA was prepared, subjected to qPCR, and analyzed using gel blots as described previously [46]. 

### 4.4. Rapid Amplification of cDNA Ends

The RACE analysis was performed using a SMARTer RACE 5′/3′ Kit (Takara Bio, Kyoto, Japan), following the manufacturer’s instructions. Briefly, the cDNA derived from the RNAs were PCR-amplified using a linker primer and *ndhA*-specific primers (Supplementary Table S2), then in-fusion cloned and sequenced.

### 4.5. Yeast Two-Hybrid Assay

The yeast two-hybrid assay was performed using the Matchmaker GAL4 two-hybrid system according to the Yeast Protocols Handbook (PT4084-1, Takara Bio, Kyoto, Japan). The *SOT1* coding region, free of its chloroplast transit peptide sequence, was fused in frame downstream of the GAL4 DNA-binding domain (BD) in a pGBKT7 vector. Likewise, the transit peptide sequences were removed from the coding region of the candidate proteins and fused to pGADT7 containing a GAL4 DNA-activation domain (AD). Briefly, the respective combinations of BD and AD fusions were cotransformed into the yeast strain Y2HGold (Takara Bio, Kyoto, Japan). The transformants were serially diluted and plated onto SD/-Trp-Leu-His-Ade dropout plates containing 40 μg mL^–1^ X-α-Gal.

### 4.6. Luciferase Complementation Assays

The assay was performed as previously described [47]. *Agrobacterium tumefaciens* strain GV3101 containing the indicated plasmids was infiltrated into expanded leaves of *N. benthamiana* and incubated in the growth room for 48 h before the Luc activity measurement. Leaf disks were then harvested and transferred into white 96 well-plates prepared in advance. Each leaf disk was incubated in the dark with 1 mM luciferase substrate for 5 min. The luminescence was recorded using a multimode plate reader (PerkinElmer EnSpire, Waltham, MA, USA). Each datapoint contains at least four replicates, and three independent experiments were carried out.

### 4.7. Coimmunoprecipitation Assay

Coimmunoprecipitation assays were performed using Arabidopsis protoplasts, as previously described by Ding et al. (2019) [46]. The *SOT1* coding sequence was cloned into the pUC19 vector containing a 3 × HA tag, and the coding sequences of *CAF1*, *CAF2*, and *Ycf4* were cloned into the pUC19 vector containing a 3 × FLAG tag. The protoplasts were transfected with the indicated plasmids, then incubated overnight. The total protein was extracted using extraction buffer containing 50 mM HEPES (pH 7.5), 150 mM KCl, 1 mM EDTA, 0.3% Triton-X 100, 1 mM DTT, and a proteinase inhibitor cocktail (Roche, Basel, Switzerland). The total protein sample was incubated with 20 μL Anti-FLAG-tag mAb-Magnetic Agarose (MBL, Minato-Ku, Japan) for 4 h and washed 5 times with extraction buffer. The bound protein was eluted with 60 μL of 0.5 mg mL^–1^ 3 × FLAG peptide (MBL, Minato-Ku, Japan) for 1 h. After elution, the proteins were separated using SDS-PAGE and detected using an anti-HA and anti-FLAG immunoblot.

### 4.8. RNA Immunoprecipitation Analysis

The RNA immunoprecipitation assay was performed as previously described [48,49]. The chloroplasts were isolated from 12-day-old seedlings or protoplasts. The intact chloroplasts were resuspended in RNA immunoprecipitation buffer (25 mM Tris-HCl (pH 8.0), 150 mM NaCl, 1 mM EDTA, 1% Triton X-100, 0.1% SDS, 0.2 unit mL^–1^ RNasin (Promega, Madison, WI, USA) and 1× protease inhibitor cocktail (Roche, Basel, Switzerland)) to extract protein–RNA complexes. The supernatant was then immunoprecipiated using antibodies against the HA tag (MBL, Minato-Ku, Japan) and GFP (Roche, Basel, Switzerland). The relative RNA-enrichment levels were determined using qPCR (for primers, see Supplementary Table S2).

### 4.9. Electrophoretic Mobility Shift Assay

EMSAs were performed using a LightShift Chemiluminescent RNA EMSA Kit (ThermoFisher, Waltham, MA, USA), following the manufacturer’s instructions. The P1−P7 RNA probes were labeled with biotin at the 3′ end using T4 RNA ligase (ThermoFisher, Waltham, MA, USA). The other RNA probes were synthesized and labeled with biotin at the 5′ end by Takara Bio. The binding reaction mixture contained 10 mM HEPES (pH 7.3), 20 mM KCl, 2 mM MgCl_2_, 1 mM DTT, 5% glycerol (*v/v*), and 1 nM RNA probes. Each protein sample was incubated with the binding reaction mixture at 22 ^o^C for 30 min. The reaction products were resolved on 7% (*w/v*) native polyacrylamide gels and transferred onto nylon membranes. The results were subsequently detected as described in the standard protocol of the chemiluminescent detection kit (ThermoFisher, Waltham, MA, USA).

## Data Availability

The data supporting the findings of this study are available within the article.

## Supplementary Materials

The following are available online at https://www.mdpi.com/article/10.3390/ijms222312639/s1.
