# Supplementary material for: CAF Proteins Help SOT1 Regulate the Stability of Chloroplast ndhA Transcripts
Source: Int J Mol Sci. 2021 Nov 23;22(23):12639. doi: 10.3390/ijms222312639 (PMC8657633; doi:10.3390/ijms222312639)
Supplement: Supplementary file 1 [file ijms-22-12639-s001.zip › ijms-1449981-supplementary.pdf]

SUPPLEMENTARY INFORMATION (SI)

CAF Proteins Help SOT1 Regulate the Stability of Chloroplast *ndhA* Transcripts

Xiuming Li [1], Wenzhen Luo [2], Wen Zhou [3], Xiaopeng Yin [2], Xuemei Wang [4], Xiujin Li [2], Chenchen Jiang [2], Qingqing Zhang [2], Xiaojing Kang [2], Aihong Zhang [2,*], Yi Zhang [2,*] and Congming Lu [2]

1 State Key Laboratory of Crop Biology, College of Horticulture Science and Engineering, Shandong Agricultural University, Taian 271018, China; lixiuming@sdau.edu.cn (X.L.)
2 State Key Laboratory of Crop Biology, College of Life Sciences, Shandong Agricultural University, Taian 271018, China; wzluo1998@163.com (W.L.); xiaopengy@126.com (X.Y.); xjli1998@126.com (X.L.); cc_jiang2019@163.com (C.J.); qingqingz2021@163.com (Q.Z.); kxj1201@163.com (X.K.); ahzhang@sdau.edu.cn (A.Z.); zhangyi@sdau.edu.cn (Y.Z.); cmlu@sdau.edu.cn (C.L.)
3 School of Life Science, Department of Biology, Southern University of Science and Technology, Shenzhen 518055, China; zhouw@sustech.edu.cn
4 Shandong Provincial Key Laboratory of Plant Stress, College of Life Sciences, Shandong Normal University, Jinan 250014, China, wangxuemei2020@sdnu.edu.cn
* Correspondence: ahzhang@sdau.edu.cn (A.Z.); zhangyi@sdau.edu.cn (Y.Z.)

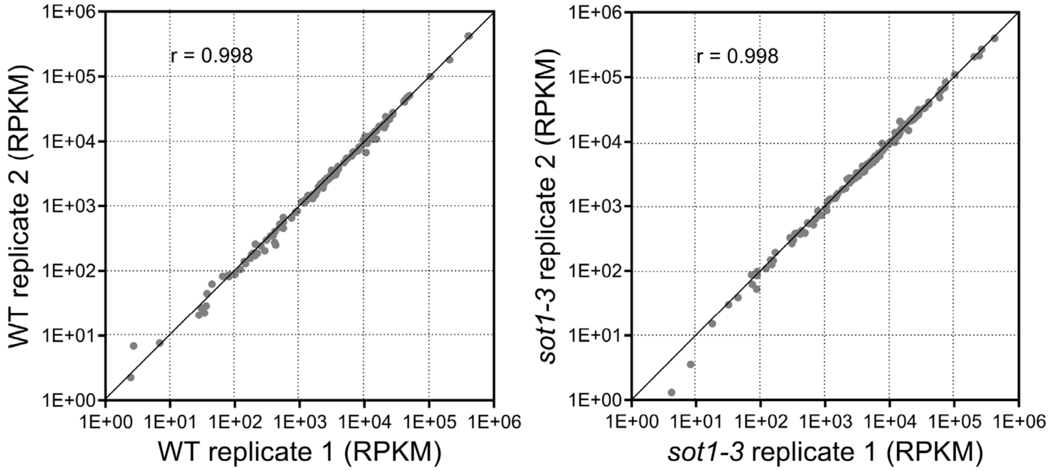

Supplementary Figure S1. Reproducibility of gene expression levels between the wild type (WT) and *sot1-3* replicates. Total RNA was isolated from 12-day-old WT and *sot1-3* seedlings and used for strand-specific RNA sequencing. The differential expression of each replicate was compared using the average number of reads per kilobase of transcript per million mapped reads (RPKM) values of total mapped reads from the chloroplast (n = 2). Genes in the large inverted repeat of the chloroplast genome were included only once.

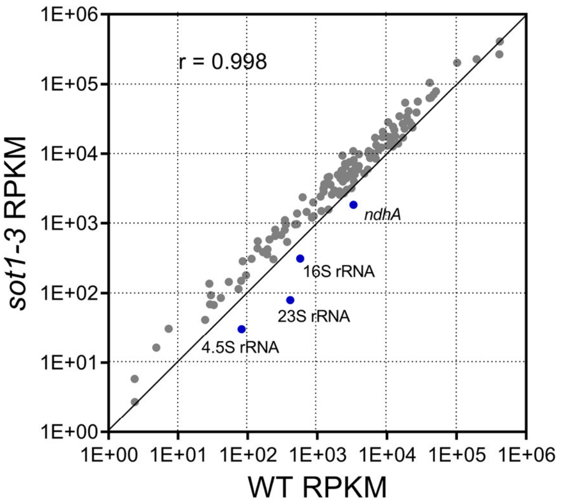

Supplementary Figure S2. Differential expression of chloroplast genes between the WT and *sot1-3* seedlings. Total RNA was isolated from 12-day-old WT and *sot1-3* seedlings and used for strand-specific RNA sequencing. The differential expression of chloroplast genes was compared between the WT and *sot1-3* using the average number of reads per kilobase of transcript per million mapped reads (RPKM) values (n = 2). Genes in the large inverted repeat of the chloroplast genome were included only once. The genes with a decreased expression in the *sot1-3* seedlings relative to the WT are highlighted in blue.

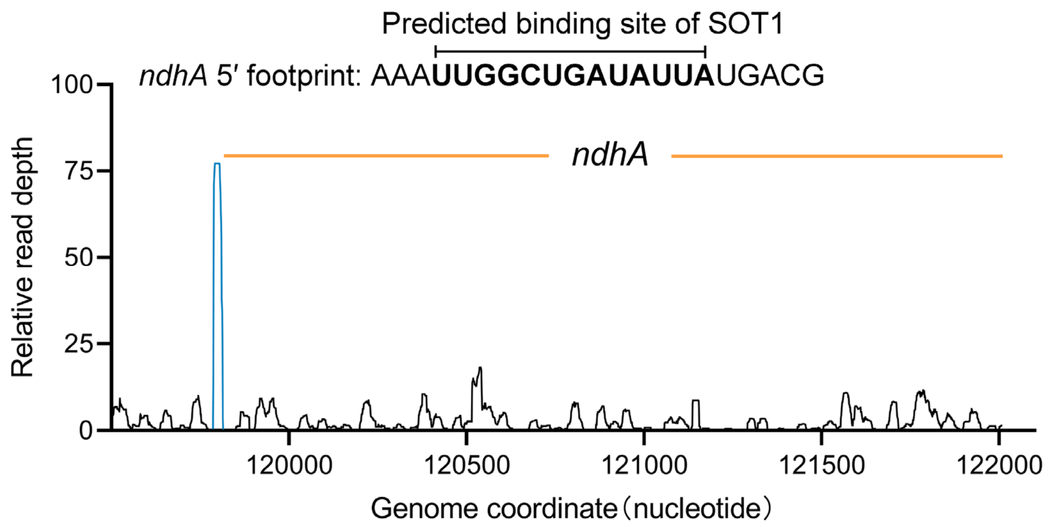

Supplementary Figure S3. Read coverage over *ndhA* regions for wild-type plants. Reads derived from small RNA sequencing were mapped to the Arabidopsis chloroplast genome. The graph indicates the read depth at each nucleotide of the *ndhA* region, normalized against the total number of reads for each sample. The RNA 'footprint' that matches the 5′-end upstream sequence of *ndhA* is highlighted in blue.

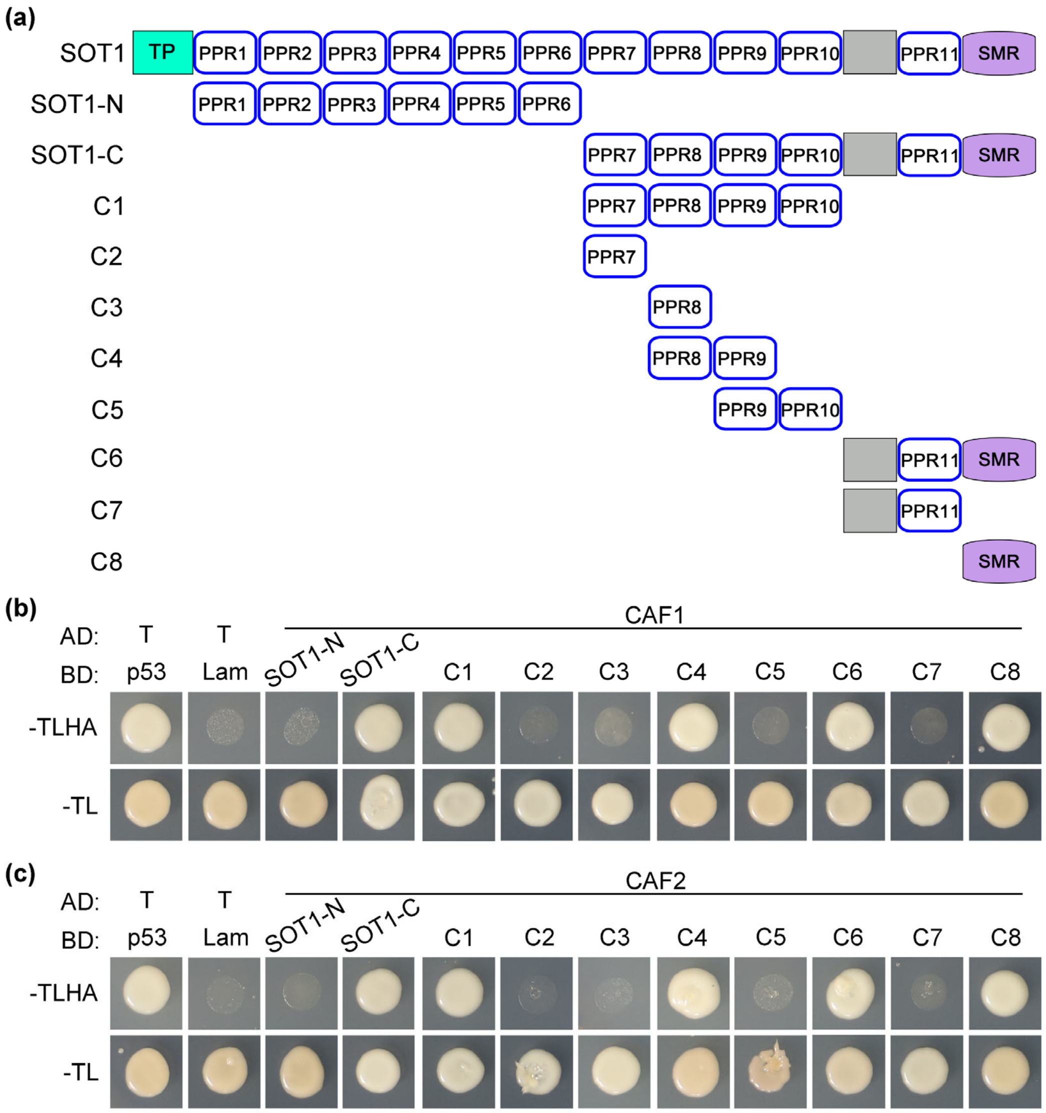

Supplementary Figure S4. Identification of the SOT1 domains that interact with CAF proteins. (**a**) Diagram the domain structures of SOT1 and various SOT1 deletions (SOT1-N, SOT1-C, and C1−C8). The turquoise and blue boxes indicate the chloroplast transit peptide (TP) and the single PPR motif (PPR1−11), respectively. The SMR domain is highlighted in purple. (**b** and **c**) Identification of the SOT1 domains that interact with (**b**) CAF1 and (**c**) CAF2 using a yeast two-hybrid assay. CAF1 and CAF2 were fused to the prey construct (AD); SOT1 and various SOT1 deletions were fused to the bait construct (BD). The ability to grow on -TLHA dropout plates indicates an interaction between the two proteins. The assay of interaction between SV40 large T-antigen (T) and murine p53 was used as a positive control; the interaction between SV40 large T-antigen (T) and lamin (Lam) was used as a negative control. -TL and -TLHA indicate SD/-Trp-Leu and SD/-Trp-Leu-His-Ade dropout plates, respectively.

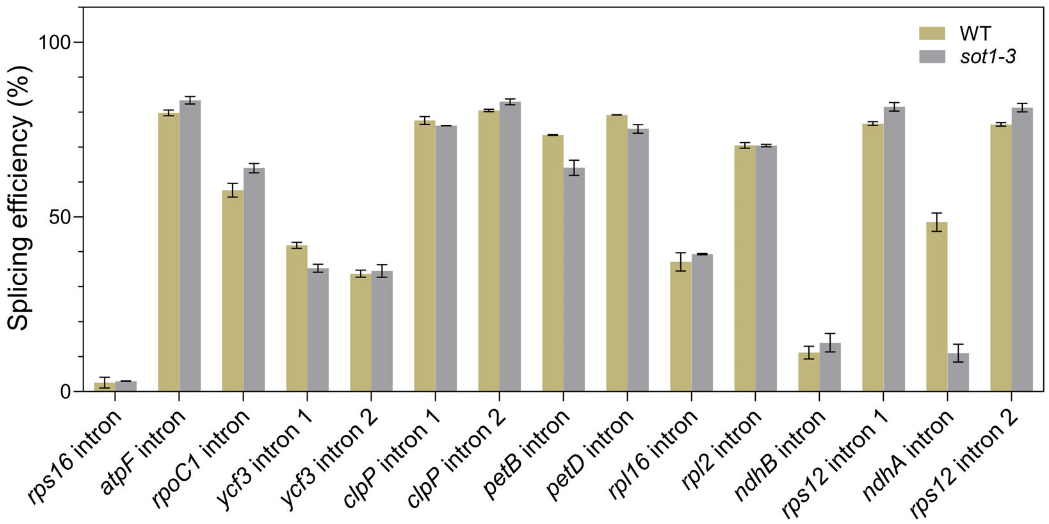

Supplementary Figure S5. RNA-sequencing (RNA-seq) determination of the RNA splicing efficiencies of chloroplast introns in 12-day-old WT and *sot1-3* seedlings.The splicing efficiency was determined according to Hotto et al. (2015). The splicing efficiency presented is the average of two replicates for both WT and *sot1-3* samples. Since the mature tRNA was discarded in the RNA-seq library preparation, the splicing efficiencies of tRNA introns was omitted. The values represent means ± SD (*n* = 2).

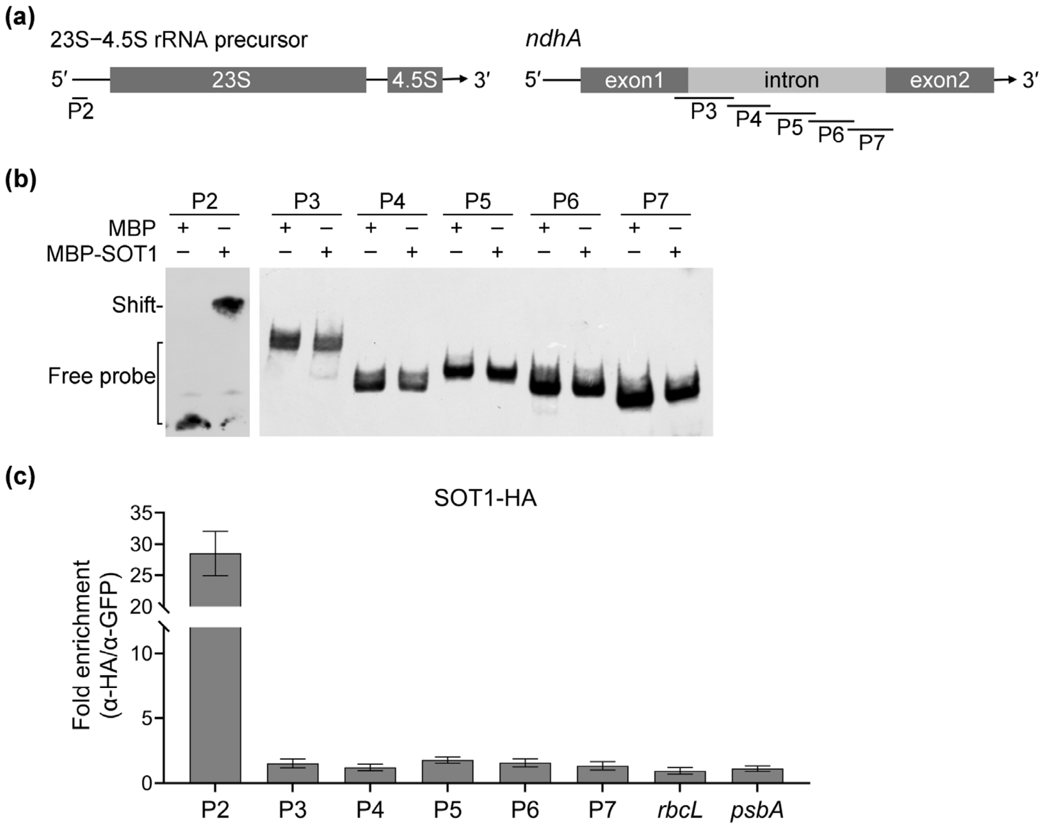

Supplementary Figure S6. SOT1 does not bind the intron of *ndhA* transcripts. (**a**) Schematic representation of the domain structures of the *ndhA* mRNA and the 23S–4.5S rRNA precursor. The positions of probes P2 to P7, subjected to RNA coimmunoprecipitation assays and electrophoretic mobility shift assay (EMSA), are shown below the models. (**b**) EMSA showing that SOT1 alone exhibits little binding activity to the *ndhA* intron. A total of 150 nM recombinant MBP and MBP-SOT1 proteins were incubated with 10 nM biotin-labeled probes. Three independent experiments were performed, and one representative experiment is shown. (**c**) RNA coimmunoprecipitation assays showing that SOT1 does not bind the *ndhA* intron in vivo. Intact chloroplasts were isolated from 12-day-old complemented *sot1-3* plants (*sot1-3/35S:SOT1-HA*). The chloroplast extracts were subjected to immunoprecipitation against HA and GFP antibodies. SOT1 was reported to bind the 5′ ends of the 23S–4.5S rRNA precursor (Wu et al., 2016; Zhou et al., 2017); therefore, this interaction served as a positive control. The sample immunoprecipitated using the GFP antibody served as a negative control. The relative RNA enrichment levels were determined using qPCR. Mean values ± SD of the triplicate replicates are shown.

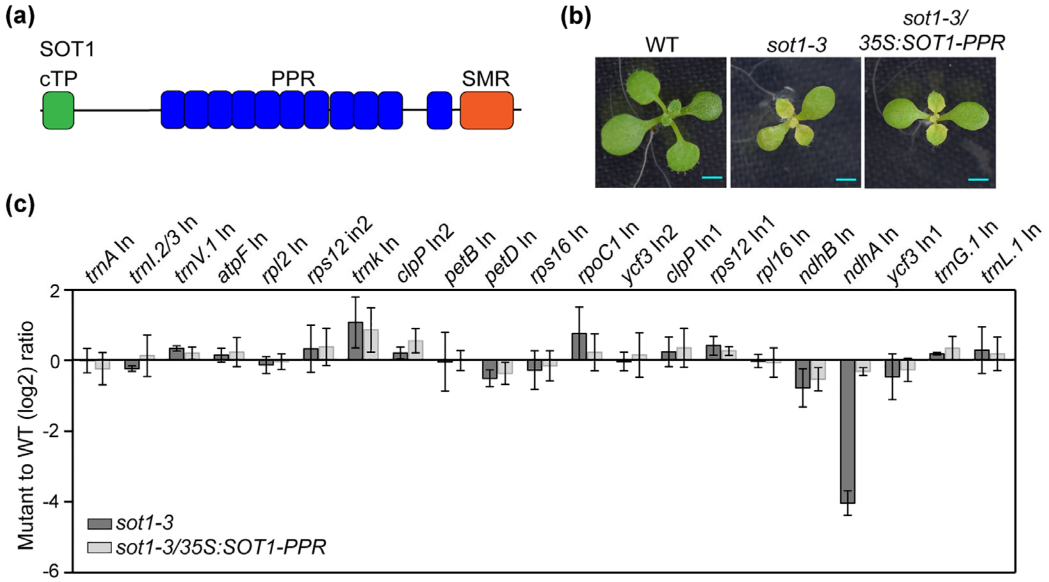

Supplementary Figure S7. The defective *ndhA* splicing in the *sot1-3* mutant was recovered in 12-day-old PPR-domain-complemented plants. (**a**) Schematic diagram of the SOT1 protein. The green and blue boxes represent the chloroplast transit peptide (TP) and each PPR motif, respectively. The SMR domain is highlighted in orange. (**b**) Phenotypes of WT, *sot1-3*, and its PPR-domain-complemented (*sot1-3/35S:SOT1-PPR*) plants grown for 12 days. (**c**) qPCR analysis of the splicing efficiency of chloroplast introns in the *sot1-3* and *sot1-3/35S:SOT1-PPR* complemented plants. The values represent the differences in the ratios of spliced to unspliced transcripts in 12-day-old *sot1-3* and *sot1-3/35S:SOT1-PPR* seedlings compared with those of the WT. Four biological replicates were analyzed. Error bars indicate SD.

Supplementary Table S1. RNA-seq alignment summary for two WT and *sot1-3* samples.

Sample	Mappable reads[a]	Mapped reads[b]	Mapped/Mappable (%)
WT rep1	47,045,476	22,527,949	47.89
WT rep2	52,133,521	23,977,993	45.99
sot1-3 rep1	51,145,460	26,044,674	50.92
sot1-3 rep2	47,618,661	22,350,101	46.94

[a]Mappable reads were selected after quality control, and therefore had a minimum length of 60 and a quality score higher than 30.

[b]Mapped reads represent the mappable reads that could be aligned to the Arabidopsis chloroplast genome (TAIR10) using TopHat2.

Supplementary Table S2. List of primers and oligonucleotides used in this study.

Purpose	Primer name	Primer sequence (5′–3′)
Quantitative PCR	*trnH*-forward	GCGGATGTAGCCAAGTGGAT
	trnH-reverse	GGCGAACGACGGGAATTGAA
	psbA-forward	GGTCGCTTCTGTAACTGGAT
	psbA-reverse	GTTGCGGTCAATAAGGTAGG
	ndhK-forward	GCCTATGGCCGCTTCTTTAT
	ndhK-reverse	GTCTAGGACTCGATCTTGGTACT
	rps16-forward	ATTGATGTTCGATCCCGAAGAG
	rps16-reverse	TCTTGTTGGTTGAGCTCCTTT
	trnQ-forward	TGGGGCGTAGCCAAGCGGTA
	trnQ-reverse	CTGGGACGGAAGGATTCGAA
	trnG-forward	TGGCGGAAATAGCTTAATGG
	trnG-reverse	AGCGGAAGGAGGGACTTGAA
	trnR-forward	GCGTCCATTGTCTAATGGAT
	trnR-reverse	TGCGTCCAATAGGATTTGAA
	trnS-forward	GGAGAGATGGCTGAGTGGAC
	trnS-reverse	GGGAAAGAGAGGGATTCGAA
	atpA-forward	TATTATTTCGAGACGTTCTG
	atpA-reverse	ATTGTATCTGTGGCTACTGC
	atpF-forward	CGATTCTTTCGTTTACTTGG
	atpF-reverse	TCATTTAACACTCCCTTTCC
	atpH-forward	CACTGGTTTCTGCTGCTTCG
	atpH-reverse	TTCTGCCTCAGGTTGTCTCG
	atpI-forward	CCTTTATTGGAACCCTGTTT
	atpI-reverse	ATCATTCGTTGGTGCTGCTA
	rps2-forward	GGGCTCGGTGTCATTATGTT
	rps2-reverse	ACGGTTGAATCCCTCTGTCT
	rpoB-forward	TTGATGTGAGGTGGGTTCAG
	rpoB-reverse	GCATATCCTGTCTAGGCAAA
	rpoC1-forward	GAGTTGAGACCCATCATTCA
	rpoC1-reverse	GTATCCACGGCTTCTTGTAC
	rpoC2-forward	CCACTCATGGTGACCTCGTT
	rpoC2-reverse	TTCGGCAGTACCTCCTGTAA
	trnC-forward	GGCGGCATGGCCGAGTGGTA
	trnC-reverse	AGGCGGCACCCGGATTTGAA
	ycf6-forward	ATGGATATAGTAAGTCTCGCAT
	ycf6-reverse	CTAGAGTCCACTTCTTCCC
	trnY-forward	GGGTCGATGCCCGAGCGGTT
	trnY-reverse	TGGGCCGAGCTGGATTTGAAC
	trnE-forward	GCCCCCATCGTCTAGTGGTT
	trnE-reverse	TACCCCCAGGGGAAGTCGAA
	trnT-forward	GCCCTTTTAACTCAGTGGTA
	trnT-reverse	AGCCCCTTATCGGATTTGAA
	psbC-forward	ACTTCTGGGACCCGAAACTC
	psbC-reverse	AAAGGCACCTACACCTAACA
	psbD-forward	CAAGGGTTTCATAATTGGAC
	psbD-reverse	ATGTATTTGCACCATCACCA
	ycf9-forward	CTTACTGATTAGTGTACCCGTTGT

	ycf9-reverse	AGGATACCCACCAAGAAGACTA
	rps14-forward	CGCGTAATAGTGCACCTACA
	rps14-reverse	CGAAGGATGTGTCCAGATAGTC
	psaA-forward	TGAGTTAGTAGCAGTGGGTG
	psaA-reverse	CAACAGTATCAATACCGTCA
	psaB-forward	ATTCGCTGGAAAGATAAACC
	psaB-reverse	ATCAAGAAAGCCGCATAAGT
	ycf3-forward	GATCTGTCATTACCGTGGAG
	ycf3-reverse	TAAGCGTTATAGCCTGTTTC
	rps4-forward	GCGTTTGGATAACATCCTTT
	rps4-reverse	GTTTGCAGCGATAACTTGGT
	trnL-forward	GGGGATATGGCGGAATTGGT
	trnL-reverse	TGGGGATAGAGGGACTTGAAC
	ndhJ-forward	TTACAAATAAAGCCCGAAGA
	ndhJ-reverse	TGATACACGCTGGCTAAGAG
	ndhC-forward	GTATCCGTGGGCAATGAGTT
	ndhC-reverse	ACCATTCCAATGCTCCTTTT
	trnV-forward	AGGGCTATAGCTCAGTTAGG
	trnV-reverse	TAGGGCTATACGGACTCGAA
	trnM-forward	ACCTACTTAACTCAGTGGTT
	trnM-reverse	TACCTACTATTGGATTTGAA
	atpE-forward	ATTGTTTGGGATTCAGAAGT
	atpE-reverse	CCATTGGTTAGCAAGGCGTA
	atpB-forward	TTGGTCTAGCGGAAACAATT
	atpB-reverse	CCTTCGCAGTAGCTTCATCG
	rbcL-forward	AAACTTGAAGGAGACAGGGAG
	rbcL-reverse	CTGAAGCCACAGGCAGAACA
	accD-forward	ATTATTGCCGAACCCTATGC
	accD-reverse	TAAAGATTCAGCCGCTTGTG
	ycf4-forward	ATATGGAAATTCGAGGTCAA
	ycf4-reverse	GGTACACGCAAGAAGTAAGC
	ycf10-forward	TGGATTACTAATTGGTGGAA
	ycf10-reverse	GTAAATTGGTTTCTGGGTAT
	petA-forward	TCGCTCCATATCTGTCTCAC
	petA-reverse	ACATACAATACGCCCAGTCG
	trnW-forward	ACGCTCTTAGTTCAGTTCGG
	trnW-reverse	CACGCTCTGTAGGATTTGAA
	trnP-forward	AGGGATGTAGCGCAGCTTGG
	trnP-reverse	TAGGGATGACAGGATTTGAA
	rpl33-forward	GTTCGAGTAACAATTATTTGG
	rpl33-reverse	TTATGCCGATTCTTTTGAGT
	rps18-forward	AATCCAAGCGATCTTTTCGT
	rps18-reverse	GTCACTCTATTCACCCGTCT
	rpl20-forward	AGCTCGGAGGCGTAGAACAA
	rpl20-reverse	CCCGATGAGCCGAAACTAAA
	rps12-forward	ACGATTAACCTCGGGATTTG
	rps12-reverse	GGTTCCTCGAACAATGTGAT
	clpP-forward	CAAAGAACGGGCAAACCTAT
	clpP-reverse	GAACCGCTACAAGATCAACA

	psbB-forward	GTTGTTGCTGGAACTATGTG
	psbB-reverse	CTTGTTGAAAGTATCCCTGA
	petB-forward	ATATGTTCCTCCGCATGTCA
	petB-reverse	ACGGTTGGACGGTAATAAAA
	petD-forward	GCTAAAGGTATGGGTCACAA
	petD-reverse	AAGCCTACGTTACAGGCAAT
	rpoA-forward	ACTCGGACACTACAGTGGAA
	rpoA-reverse	AAGTAAAGCTCTTCGCATCG
	rps11-forward	GGTCGGGTGATTTCTTGGTC
	rps11-reverse	CAGCTCGTTGCATACCTTGATC
	rpl36-forward	AAATAAGGGCTTCCGTTCGT
	rpl36-reverse	TTTGTTTATGCCTCGGGTTG
	rps8-forward	CGACCGGGTCTACGAATCTA
	rps8-reverse	GCTTCTCGGTCTGTCATTATACC
	rpl14-forward	TAGTAATCGCCGATATGCTC
	rpl14-reverse	GTCCCATTGTTACGTTTGAG
	rpl16-forward	CGCAATGACACGAAATGTAC
	rpl16-reverse	TCCTTTCCCAGAACCCATAC
	rpl22-forward	TATGCCTTATCGAGGATGTT
	rpl22-reverse	AGTATTCCCTTGGTTCACCT
	rps19-forward	AGAAATCATAATAACTTGGTCC
	rps19-reverse	TATAAACGGGTAAGTGTTCC
	rpl2-forward	GCTGTAGCGAAACTGATTGC
	rpl2-reverse	ACTTGTCCGACTGTTGCTGA
	rpl23-forward	GGGTCGAACTCTTCTTTGGT
	rpl23-reverse	AACCCGGTTGAAGCGTAATG
	trnI-forward	GGGCTATTAGCTCAGTGGTA
	trnI-reverse	TGGGCCATCCTGGATTTGAA
	ycf2-forward	ACCGATTCCCTAAATACCTT
	ycf2-reverse	GAAATTCTTCGCTTTCTTCC
	orf77-forward	ATGCTACTACTGAAACATGGAAGA
	orf77-reverse	GTAGTGAGTAATAGCTCCGGTTG
	ndhB-forward	CTCCCACTCCAGTCGTTGCT
	ndhB-reverse	TCCAGAAGAAGATGCCATTC
	rps7-forward	TAGGCGGGTCAACTCATCAA
	rps7-reverse	CCTTTGGCAGCATCCACTAA
	rrn16S-forward	CGCTAGTAATCGCCGGTCAG
	rrn16S-reverse	CCTCCTTGCGGTTAAGGTAA
	trnA-forward	GGGGATATAGCTCAGTTGGT
	trnA-reverse	TGGAGATAAGCGGACTCGAA
	trnL-forward	GGGGATATGGCGGAATTGGT
	trnL-reverse	TGGGGATAGAGGGACTTGAAC
	rrn23S-forward	TGGGCGTTAGAGCATTGAGA
	rrn23S-reverse	GTTATCCGCTCCGCACTTGG
	rrn4.5S-forward	GAAGGTCACGGCGAGACGAGCC
	rrn4.5S-reverse	GTTCAAGTCTACCGGTCTGTT
	rrn5S-forward	TCCTCAGTAGCTCAGTGGTA
	rrn5S-reverse	CTCCCCAAGTAGGATTCGAA
	trnN-forward	CGGGTTATGGACGAAGGAGA

	trnN-reverse	CCAATGCTAAATGCAGAGGC
	ycf1-forward	TACTATGGTAGCGGCGGGTAT
	ycf1-reverse	TGAGCAAGAGCTAAAGTGGC
	ndhF-forward	AAAACGTACTTCTATCTCGA
	ndhF-reverse	TGTAGAAAGTGATTTCCCTA
	rpl32-forward	TCCCAGTGGTTAATGATGCA
	rpl32-reverse	CCGACTTTGCGAAATGTAAT
	ycf5-forward	TCCTCAGTAGCTCAGTGGTA
	ycf5-reverse	CTCCCCAAGTAGGATTCGAA
	ndhD-forward	TTCTTCTAACGACCTACGCT
	ndhD-reverse	TCTATTCCCATTCTCCAGTA
	psaC-forward	CTTGTTTTGAGTGCCTATTT
	psaC-reverse	AACTGCATTGAGTATAAGTT
	ndhE-forward	GCTGCTGCACAACTCCTTAT
	ndhE-reverse	CAAACCAACGAAGTAATCCC
	ndhG-forward	CTGTAACTATTCAATATCCCTA
	ndhG-reverse	GTTTCCAATTTCCAATCAAC
	ndhI-forward	TTTACCAGAAGCGGAAGAAG
	ndhI-reverse	CAACCGCCTAAGTATAGAAC
	ndhA-forward	TCGTATTGGAGGAATAGCGG
	ndhA-reverse	GGATTTCGTGTAATGAGTTT
	ndhH-forward	TTCACTAATAAGATACGAAGAC
	ndhH-reverse	TTTTGACAAATAAGCCAGCA
	rps15-forward	CTTGTTTTGAGTGCCTATTT
	rps15-reverse	AACTGCATTGAGTATAAGTT
RNA gel blot	probe a-forward	GGATCTTCGTAAAATTGATCG
	probe a-reverse	ATCTATGCTACCGAGTATCGTC
	probe b-forward	AAGTGAGCGGCAATTAGGTAA
	probe b-reverse	CCCGAATTATACCAATGGAATTC
	probe c-forward	GTACAGTTGATATAGTTGAGGC
	probe c-reverse	AGATTACCAAGGGAAATAGG
	probe d-forward	ATGCTTCCTATGATAACCGG
	probe d-reverse	TCAAATTAAAGGGTTTACCCC
	probe e-forward	ATGGATTTGCCTGGACCAA
	probe e-reverse	GACGAGCCACAGAAATTGC
	probe f-forward	ATGATACTCGAACATGTACTTGT
	probe f-reverse	CTTATTTAATAAGGTCGATTGG
	probe g-forward	GTCACATTCAGTAAAAATTTATG
	probe g-reverse	TCAATAAGCTAGACCCATACT
	probe h-forward	AGCAATGTACAGCGGTCAAA
	probe h-reverse	ATTCGAACCAACCCATAGGC
RACE	*ndhA* 5′	CCTGACATAAGAAGTCCAATAGGAGC
qRT-PCR for splicing	*trnA*(unspliced)-forward	GGGGATATAGCTCAGTTG
	trnA(spliced)-reverse	TGGAGATAAGCGGACTC
	trnA(unspliced)-reverse	TAGAAAAAGTGAGCCACC
	trnI(spliced)-forward	GGGCTATTAGCTCAGTGGTAG
	trnI(unspliced)-reverse	TGGGCCATCCTGGATTTG
	trnI(unspliced)-forward	CGTTCGGGAAGGATGAATC
	trnV(unspliced)-forward	AGGGCTATAGCTCAGTTAG

	trnV(spliced)-reverse	TAGGGCTATACGGACTC
	trnV(unspliced)-reverse	GACATCGATTTCTTAATAAGATC
	atpF(spliced)-forward	GCAACAAATCCAATAAATCTAAG
	atpF(unspliced)-reverse	ATAGCTCCTTCACGCAG
	atpF(unspliced)-forward	TTCGGGAAGGGATCATAG
	rpl2(spliced)-forward	CAGAGGGGCTATAATTGGAG
	rpl2(unspliced)-reverse	CGCTGCTCTAGCTAATTG
	rpl2(unspliced)-forward	TGCTTTGGAAGAAGCTTG
	rps12-2(unspliced)-forward	CTGTAGTCTTAGTAAGAGGG
	rps12-2(spliced)-reverse	TTATTTTGGCTTTTTGACCC
	rps12-2(unspliced)-reverse	CTTGGATAAGAATCTACAACG
	trnK(unspliced)-forward	GGGTTGCTAACTCAACG
	trnK(spliced)-reverse	ACTCGAACCCGGAACTAG
	trnK(unspliced)-reverse	GTGGTCTTACAAACTCTACC
	clpP-2(unspliced)-forward	TTCAATAGCATCCTTTATCC
	clpP-2(spliced)-reverse	AAGATAAATTCTCCCGTTTG
	clpP-2(unspliced)-reverse	CTAATTCATATTTCAAATGGCG
	petB(spliced)-forward	CATTGTATATTTCCGGAATATGAG
	petB(unspliced)-reverse	TATGTTGACATGCGGAGGAA
	petB(unspliced)-forward	TCTTGGAGGGGGAGTAACCT
	petD(spliced)-forward	GAAGAGATAATGGATTATGGGAGTG
	petD(unspliced)-reverse	GGGTTCCCCGTAATAATTGTG
	petD(unspliced)-forward	AAAAATTATCATGTCCGGTTCC
	rps16(spliced)-forward	ATGGTAAAACTTCGTTTAAAAC
	rps16(unspliced)-reverse	TAAGATCTCTTCCTTCTCTTCG
	rps16(unspliced)-forward	CCGTACGAGGCCAAAAC
	rpoC1(spliced)-forward	CGTCTTCCTAGTTATATTGC
	rpoC1(unspliced)-reverse	ACCTCGTAATCGTAAGAAAG
	rpoC1(unspliced)-forward	GGATGAGAGGAAACTTTCATG
	ycf3-2(spliced)-forward	TTCGGGCATTAGAACGAAAC
	ycf3-2(unspliced)-reverse	AATACTCAGCGGCTTGAG
	ycf3-2(unspliced)-forward	TGAGGTAGGAAACTCTCAAGTAC
	clpP-1(unspliced)-forward	CCTATTGGCGTTCCAAAAG
	clpP-1(spliced)-reverse	ATTCGAGATTTCGGTATCAACC
	clpP-1(unspliced)-reverse	TGGGGAAATCCCATATAGC
	rps12-1(unspliced)-forward	AGAAATACAAGACAGCCAATC
	rps12-1(spliced)-reverse	TACGTAAAGCAGAGTTTGG
	rps12-1(unspliced)-reverse	TCAGTCTATGATCTAAACGAGTC
	rpl16(spliced)-forward	TCCTTTGATATAATTGCTATGCTTAGT
	rpl16(unspliced)-reverse	CCAAATTTTTCCACCACGTC
	rpl16(unspliced)-forward	GAAACTCTCACGTTCAGTTCTGT
	ndhB(spliced)-forward	TCATCAATGGACTCCTGACG
	ndhB(unspliced)-reverse	CCAGAAGAAGATGCCATTCA
	ndhB(unspliced)-forward	AGTCTCATGCACGGTTTTGA
	ndhA(spliced)-forward	CTGCCCAATCGATTAGTTATG
	ndhA(spliced)-reverse	GTTTTTGGGGATGGAATTTG
	ndhA(unspliced)-forward	TGAGGCCAAGACCTCATG

	ycf3-1(spliced)-forward	CGAGTCATTCCGACAACTTC
	ycf3-1(unspliced)-reverse	TGTGGTAAAAAGGGGTTTCG
	ycf3-1(unspliced)-forward	CCTAAAGGAGGAGCCGTATG
	trnG(unspliced)-forward	GCGGGTATAGTTTAGTGG
	trnG(spliced)-reverse	GGTAGCGGGAATCGAAC
	trnG(unspliced)-reverse	GGAAAGGACTAAATCCTTTTAAG
	trnL(spliced)-forward	GGGATATGGCGGAATTG
	trnL(unspliced)-reverse	TGGGGATAGAGGGACTTG
	trnL(unspliced)-forward	GATAGAGTCCCATTTTACATGTC
RT-PCR for splicing	*trnA*-RT-forward	GGGGATATAGCTCAGTTG
	trnA-RT-reverse	TGGAGATAAGCGGACTC
	trnI-RT-forward	GGGCTATTAGCTCAGTGGTAG
	trnI-RT-reverse	TGGGCCATCCTGGATTTG
	trnV-RT-forward	AGGGCTATAGCTCAGTTAG
	trnV-RT-reverse	TAGGGCTATACGGACTC
	atpF-RT-forward	GCAACAAATCCAATAAATCTAAG
	atpF-RT-reverse	ATAGCTCCTTCACGCAG
	rpl2-RT-forward	CAGAGGGGCTATAATTGGAG
	rpl2-RT-reverse	CGCTGCTCTAGCTAATTG
	rps12-2-RT-forward	CTGTAGTCTTAGTAAGAGGG
	rps12-2-RT-reverse	TTATTTTGGCTTTTTGACCC
	clpP-2-RT-forward	TTCAATAGCATCCTTTATCC
	clpP-2-RT-reverse	AAGATAAATTCTCCCGTTTG
	petB-RT-forward	CATTGTATATTTCCGGAATATGAG
	petB-RT-reverse	TATGTTGACATGCGGAGGAA
	petD-RT-forward	GAAGAGATAATGGATTATGGGAGTG
	petD-RT-reverse	GGGTTCCCCGTAATAATTGTG
	rps16-RT-forward	ATGGTAAAACTTCGTTTAAAAC
	rps16-RT-reverse	TAAGATCTCTTCCTTCTCTTCG
	rpoC1-RT-forward	CGTCTTCCTAGTTATATTGC
	rpoC1-RT-reverse	ACCTCGTAATCGTAAGAAAG
	ycf3-2-RT-forward	TTCGGGCATTAGAACGAAAC
	ycf3-2-RT-reverse	AATACTCAGCGGCTTGAG
	clpP-1-RT-forward	CCTATTGGCGTTCCAAAAG
	clpP-1-RT-reverse	ATTCGAGATTTCGGTATCAACC
	rps12-1-RT-forward	AGAAATACAAGACAGCCAATC
	rps12-1-RT-reverse	TACGTAAAGCAGAGTTTGG
	rpl16-RT-forward	TCCTTTGATATAATTGCTATGCTTAGT
	rpl16-RT-reverse	CCAAATTTTTCCACCACGTC
	ndhB-RT-forward	TCATCAATGGACTCCTGACG
	ndhB-RT-reverse	CCAGAAGAAGATGCCATTCA
	ndhA-RT-forward	CTGCCCAATCGATTAGTTATG
	ndhA-RT-reverse	GTTTTTGGGGATGGAATTTG
	ycf3-1-RT-forward	CGAGTCATTCCGACAACTTC
	ycf3-1-RT-reverse	TGTGGTAAAAAGGGGTTTCG
	trnG-RT-forward	GCGGGTATAGTTTAGTGG
	trnG-RT-reverse	GGTAGCGGGAATCGAAC
	trnL-RT-forward	GGGATATGGCGGAATTG
	trnL-RT-reverse	TGGGGATAGAGGGACTTG

Poisoned primer extension analysis	PPE(*ndhA*)-reverse	AACTGTACTTAAACTGTTAGAT(ddCTP)
	PPE(*clpP* Intron2)-reverse	GCGGGTTGATGGATCATTACCC(ddCTP)
	PPE(*rps12* Intron1) -reverse	AGCAGAGTTTGGTTTTTTGGGGG(ddCTP)
Y2H assay	CAF1(AD:EcoRI) -forward	GGAATTCCATATGGCCGAACGGTTCGAG CAGCG
	CAF1(AD:BamHI) -reverse	CGCGGATCCTGCAAGTAGTTTAGCTAGTT C
	CAF2(AD:EcoRI) -forward	GGAATTCTCCAATAGAAATCGTAATC
	CAF2(AD:BamHI) -reverse	CGCGGATCCTTTTAGTCTTTCCCTCAACTT C
	CFM2(AD:SmaI) -forward	TCCCCCGGGTTGTAGTTCTGCTTCCGGTC
	CFM2(AD:BamHI) -reverse	CGCGGATCCCAAACCACATTCAAGTCTT ATGG
	CRS2(AD:EcoRI) -forward	GGAATTCCATATGGCTTCGTTGCCTGTTT CTG
	CRS2(AD:BamHI) -reverse	CGCGGATCCAACTTTATGAAACTTGTATT TCTGTC
	SOT1(BD:NcoI) -forward	CATGCCATGGAGTCCTTGAAACAACCTA AAACC
	SOT1(BD:BamHI) -reverse	CGCGGATCCTGCTTGAGAAGTAACTAAA GG
Luciferase Complementati -on assays	CAF1(CLuc:KpnI) -forward	GGGGTACCATGTCGTTAAAACTCAACAC TCC
	CAF1(CLuc:SalI) -reverse	ACGCGTCGACTGCAAGTAGTTTAGCTAG TTCATC
	CAF2(CLuc:KpnI) -forward	GGGGTACCATGGCGATTGTAGCATCACT AA
	CAF2(CLuc:SalI) -reverse	ACGCGTCGACTTTTAGTCTTTCCCTCAAC TTCC
	CFM2(CLuc:KpnI) -forward	GGGGTACCATGTTGCTTCCACTGTTTC
	CFM2(CLuc:SalI) -reverse	ACGCGTCGACCAAACCACATTCAAGTCT TA TG
	CRS2(CLuc:KpnI) -forward	GGGGTACCATGGTTACAGCGATGTTTTG
	CRS2(CLuc:SalI) -reverse	ACGCGTCGACAACTTTATGAAACTTGTAT TTCTG
	CRS2(NE:KpnI) -reverse	CGGGGTACCAACTTTATGAAACTTGTATT TCTG
	SOT1(NLuc:KpnI) -forward	GGGGTACCATGGCGACTGTTCTTACCAC
	SOT1(NLuc:SalI) -reverse	ACGCGTCGACTGCTTGAGAAGTAACTAA AGG
Co-IP assay	CAF1(pUC19:KpnI) -forward	GGGGTACCATGTCGTTAAAACTCAACAC TCC
	CAF1(pUC19:BstBI) -reverse	ACTTCGAATGCAAGTAGTTTAGCTAGTTC ATC

	CAF2(pUC19:KpnI)-forward	GGGGTACCATGGCGATTGTAGCATCAC
	CAF2(pUC19:BstBI)-reverse	ACTTCGAATTTTAGTCTTTCCCTCAACTTCC
	YCF4(pUC19:KpnI)-forward	GGGGTACCATGAGTTGGCGATCAGAATCTATATG
	YCF4(pUC19:BstBI)-reverse	GCTTCGAAAAATACTTCAATTGGTACACGC
	SOT1(pUC19:KpnI)-forward	GGGGTACCATGGCGACTGTTCTTACCAC
	SOT1(pUC19:SalI)-reverse	ACGCGTCGACTGCTTGAGAAGTAACTAAAGG
EMSA	probe P1-forward	AGGAGATCAAAGCGGTTTT
	probe P1-reverse	TTTAACGATCAACTTCTCCC
	probe P2-forward	CTCGTGAACTTAGCCGATAC
	probe P2-reverse	TGGAAAGATCTTATCAACGT
	probe P3-forward	CTGCCCAATCGATTAGTTAT
	probe P3-reverse	CCGTTTAACCTTTGTACATAGG
	probe P4-forward	CCTATGTACAAAGGTTAAACGG
	probe P4-reverse	CCTTTTCAATTTGGTTAATCTC
	probe P5-forward	GAGATTAACCAAATTGAAAAGG
	probe P5-reverse	CTTGTTTCGTTCCTATTCTTC
	probe P6-forward	GAAGAATAGGAACGAAACAAG
	probe P6-reverse	CTGTCTGCTCAAATTCTAAGA
	probe P7-forward	TCTTAGAATTTGAGCAGACAG
	probe P7-reverse	GCCTCAACTATATCAACTGTAC
	RNA 12	Biotin-UGGCUGAUAUUA
